# α-Ketoglutarate Promotes Hepatic Steatosis by Enhancing Cidec Stability via Lactylation–Ubiquitination Crosstalk

**DOI:** 10.34133/research.1471

**Published:** 2026-11-09

**Authors:** Rui Yin, Liangli Dai, Hongqiu Zhang, Weijian Hang, Lu He, Xiwei Kang, Junfang Wu, Anbing Shi, Yugang Wang, Yumei Wang, Peng Zhang, Juan Chen

**Affiliations:** ^1^ Department of Biochemistry and Molecular Biology, School of Basic Medicine, Tongji Medical College, Huazhong University of Science and Technology, Wuhan, Hubei, China.; ^2^ Department of Internal Medicine, Division of Cardiology, Tongji Hospital, Tongji Medical College, Huazhong University of Science and Technology, Wuhan, Hubei, China.; ^3^ Faculty of Arts and Science, University of Toronto, Toronto, ON, Canada.; ^4^ Department of Nephrology, Union Hospital, Tongji Medical College, Huazhong University of Science and Technology, Wuhan, Hubei, China.; ^5^ Hubei Key Laboratory of Natural Active Polysaccharides, Union Hospital, Tongji Medical College, Huazhong University of Science and Technology, Wuhan, Hubei, China; ^6^ Taikang Medical School (School of Basic Medical Sciences), Wuhan University, Wuhan, Hubei, China.

## Abstract

α-Ketoglutarate (AKG), a key tricarboxylic acid cycle intermediate, is widely consumed as a dietary supplement by healthy individuals for its perceived anti-aging and performance-enhancing benefits. While its antidiabetic and anti-obesity effects have been reported under pathological conditions, the safety profile and mechanisms of long-term AKG intake in healthy animals remain largely unexplored. Here, we demonstrate that long-term AKG intake via food or drinking water markedly elevates hepatic triglyceride levels and induces steatosis in healthy mice by suppressing adipose triglyceride lipase (Atgl) activity. The lipid-droplet-associated protein, cell death-inducing DNA fragmentation factor 45-like effector C (Cidec), is an inhibitor of Atgl. We find that AKG stabilizes Cidec by attenuating tripartite-motif protein 21-mediated ubiquitination. Mechanistically, AKG reduces H3K4me3 at the *Pck1* promoter, activating *Pck1* transcription, which promotes lactate production and subsequent Cidec lactylation. This lactylation inhibits Cidec ubiquitination, restrains Atgl activity, and ultimately drives hepatic triglyceride accumulation. Hepatocyte-specific knockdown of *Cidec* or *Pck1* markedly ameliorates AKG-induced steatosis. Collectively, our findings reveal that long-term AKG intake enhances Cidec stability through a phosphoenolpyruvate carboxykinase 1- and tripartite-motif protein 21-mediated “lactylation–ubiquitination crosstalk”, thereby inhibiting Atgl activity and contributing to hepatic steatosis in mice. These results caution that even AKG with established health benefits may pose a risk of liver injury when used chronically in healthy mice.

## Introduction

α-Ketoglutarate (AKG) is a key intermediate metabolite in the tricarboxylic acid (TCA) cycle that plays a critical role in various physiological processes, including energy metabolism, amino acid metabolism, and redox regulation [1,2]. In the TCA cycle, AKG is produced via the dehydrogenation of isocitrate and then converted into succinyl-CoA through the catalysis of the AKG dehydrogenase complex, generating nicotinamide adenine dinucleotide and providing electrons for the oxidative phosphorylation process [3]. Recent studies have shown that AKG plays a significant role in tumor suppression, anti-aging, and metabolic modulation [4–6].

As an intermediate metabolite of the TCA cycle, AKG takes part in regulating cellular energy metabolism. Cheng et al. [7] reported that AKG supplementation significantly improved hyperlipidemia-induced liver steatosis. Yuan et al. [6] proposed that AKG supplementation significantly reduces the high-fat-diet-induced increase in blood glucose. Tian et al. [8] reported that AKG supplementation significantly reduced high-fat diet-induced weight gain. Collectively, recent reports regarding AKG and metabolic regulation have focused mainly on pathological conditions [6–8], and the mechanisms of AKG in healthy individuals remain unclear. Currently, AKG is used as a dietary supplement, and its consumers may not necessarily have pathological conditions. Therefore, it is of great significance to investigate the effects and underlying mechanisms of AKG in healthy individuals. As AKG has a direct involvement in the TCA cycle, long-term AKG intake may provide additional energy and increase the metabolic burden on the liver. Therefore, we aimed to investigate the metabolic effects of prolonged AKG consumption in healthy individuals.

Here, we investigated the long-term effects of AKG consumption by supplementing drinking water or the diet with 2% AKG for 24 weeks. Surprisingly, long-term AKG intake led to liver steatosis, increased liver weight, and impaired liver function in healthy mice. Mechanistically, long-term AKG feeding promoted the expression of cell death-inducing DNA fragmentation factor 45-like effector C (Cidec) by increasing its protein stability. Cidec inhibits the lipase activity of adipose triglyceride lipase (Atgl), leading to triglyceride (TG) accumulation in hepatocytes. Interestingly, long-term AKG treatment suppresses the proteasomal degradation of Cidec through “lactylation–ubiquitination crosstalk” mediated by phosphoenolpyruvate carboxykinase 1 (Pck1) and tripartite-motif protein 21 (Trim21).

## Results

### Long-term AKG intake promotes hepatic steatosis

It has been reported that the addition of 2% AKG to drinking water for 2 weeks effectively alleviated obesity but did not affect systemic metabolism in normal mice [6]. To evaluate the effects of long-term AKG intake on systemic metabolism, we provided the mice drinking water supplemented with 2% AKG for 24 weeks. AKG consumption did not affect the body weights or serum TG or glucose levels of the mice compared with those in the control group (Fig. 1A to C). AKG administration significantly increased the AKG content in the serum of mice (Fig. 1D). White adipose tissue and the liver are key organs related to lipid metabolism [9]. AKG consumption did not change the epididymal white adipose tissue (eWAT) weight or eWAT-to-body weight ratio in the mice (Fig. 1E and F). Surprisingly, liver weights and liver-to-body weight ratios were significantly increased by the 24th week of AKG consumption, but liver weights and liver-to-body weight ratios were not changed in the 4th week (Fig. 1G and H). TG levels also significantly increased in the 24th week, whereas there was no change in the 4th week. (Fig. 1I). Aspartate transaminase (AST) is a biomarker indicative of hepatic injury. Surprisingly, the serum AST and alanine aminotransferase (ALT) concentration significantly increased at the 24th week but not at the 4th week (Fig. 1J and K). Oil Red O staining of liver tissue sections revealed a significant increase in hepatic lipid accumulation after the 24th week of AKG consumption but not after the 4th week (Fig. 1L). AKG supplementation did not alter food or water intake in mice (Fig. 1M and N).

**Fig. 1.**
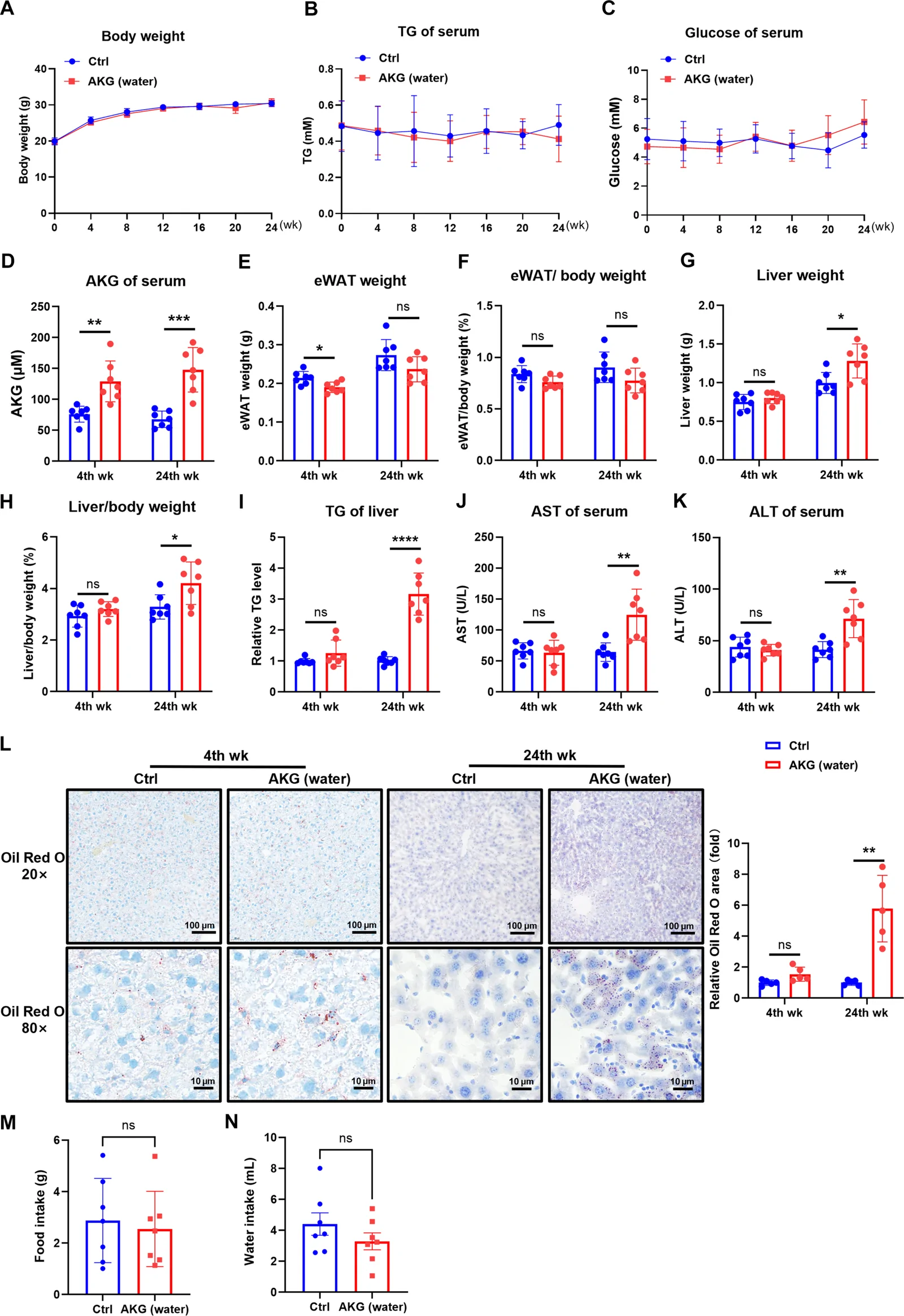
Long-term α-ketoglutarate (AKG) intake through drinking water promotes hepatic lipid accumulation. (A) Body weight was monitored every month in C57BL/6 mice supplemented with 2% AKG via drinking water (*n* = 7). (B) Serum triglyceride (TG) levels (*n* = 7). (C) Blood glucose levels were measured monthly (*n* = 7). (D) Serum AKG level in mice at the 4th or 24th week of AKG feeding (*n* = 7). (E) The weight of epididymal white adipose tissue (eWAT) in mice at 4th or 24th week of AKG feeding (*n* = 7). (F) The percentage of eWAT relative to total body weight (*n* = 7). (G) The weight of the liver in mice (*n* = 7). (H) The percentage of liver weight relative to total body weight (*n* = 7). (I) Hepatic TG levels measured using biochemical kits (*n* = 7). (J) Serum aspartate transaminase (AST) levels were measured monthly (*n* = 7). (K) Serum alanine aminotransferase (ALT) levels were measured monthly (*n* = 7). (L) Oil Red O staining of liver tissues from mice supplemented with 2% AKG via drinking water (*n* = 5). (M) Daily food intake per mouse at week 24 (*n* = 7). (N) Daily water intake per mouse at week 24 (*n* = 7). Data are presented as mean ± standard deviation. *P < 0.05, **P < 0.01, ***P < 0.001, ****P < 0.0001; ns, not significant. *P* values were determined using an unpaired Student *t* test (A to N).

To avoid the confounding factor of AKG intake method, we conducted animal experiments in which 0.1%, 1%, or 2% AKG was added to food (Figs. S1 and S2). Like AKG supplementation via drinking water, AKG supplementation via food did not affect the body weights of the mice (Figs. S1A and S2A). Interestingly, serum TG levels significantly increased in the 16th week and continued to rise thereafter (Figs. S1B and S2B), while blood glucose levels were not affected by 1% and 2% AKG consumption (Figs. S1C and S2C). Also, 1% or 2% AKG did not affect glucose tolerance or insulin sensitivity in mice (Figs. S1D to G and S2D to G). AKG administration significantly increased the AKG content in the serum of mice (Figs. S1H and S2H). Compared with those of the control group, eWAT weights and eWAT-to-body weight ratios were not significantly different at either the 4th or 24th week of 1% or 2% AKG feeding (Figs. S1I and J and S2I and J). In the fourth week of 1% or 2% AKG feeding, liver weights, liver-to-body weight ratios, and hepatic TG levels did not significantly change (Figs. S1K to M and S2K to M). Importantly, by the 24th week of 1% and 2% AKG feeding, liver weights, liver-to-body weight ratios, and hepatic TG levels were significantly increased (Figs. S1K to M and S2K to M). By the 24th week of 1% and 2% AKG feeding, serum AST and ALT levels significantly increased (Figs. S1N and O and S2N and O). Oil Red O staining of liver tissue sections revealed a marked increase in hepatic steatosis after 24 weeks of 1% and 2% AKG feeding but not at the 4 weeks (Figs. S1P and S2P). However, 0.1% AKG feeding did not alter serum TGs, liver weight, liver weight-to-body weight ratio, hepatic steatosis, or liver function in mice (Fig. S2B to P).

Hepatocytes are the predominant parenchymal cells in the liver and are responsible for nearly all hepatic metabolic functions. Therefore, we hypothesized that hepatocytes are the primary target cells of AKG. First, we treated AML12 cells and primary hepatocytes with different concentrations of AKG for 24 h. Significant cellular damage was observed in AML12 cells in response to 500 μM AKG and in primary hepatocytes at 100 μM AKG (Fig. S3A and B). Therefore, we used 500 μM AKG to determine the effects of AKG treatment on hepatocytes at different time points. Within 12 h, AKG caused no significant cytotoxicity in AML12 cells or primary hepatocytes (Fig. S3C and D). However, after 18 h, AKG markedly reduced the viability of both cell lines (Fig. S3C and D). In the AML12 cell line and in primary hepatocytes, TG levels significantly increased after 24 h of AKG treatment, whereas no significant changes were observed after short-term treatment (6 h) (Fig. S3E and F). Total cholesterol (TC) levels in AML12 cells and primary hepatocytes did not significantly change (Fig. S3G and H). Most of the TGs within cells are stored in lipid droplets, which can be observed using boron dipyrromethene (BODIPY) staining followed by laser confocal microscopy. After 24 h of AKG treatment, the area of BODIPY-labeled lipid droplets significantly increased in both AML12 cells and primary hepatocytes (Fig. S3I and J). However, the area of lipid droplets in hepatocytes did not significantly change after 6 h of AKG treatment (Fig. S3I and J).

These results indicate that long-term, rather than short-term, AKG treatment significantly increased the TG contents in mouse hepatocytes.

### Down-regulated Atgl activity leads to TG accumulation in AKG-treated hepatocytes

Fatty acids are essential substrates for TG synthesis [10]. To investigate the mechanism by which AKG induces TG accumulation in hepatocytes, we first examined whether AKG affects TG levels via fatty acid metabolism. We examined the expression of proteins related to fatty acid uptake (Cd36), synthesis (Fasn, ACC1), and β-oxidation (Cpt1a) and found that none were affected by AKG treatment (Fig. 2A and Fig. S4A). Furthermore, we assessed the enzymatic activities of Fasn and fatty acid β-oxidation (FAO). Interestingly, while Fasn activity remained unchanged, FAO activity was increased at both 6 and 24 h following AKG treatment, which was inconsistent with the increased TG contents (Fig. 2B and C and Fig. S4B and C). These results suggest that AKG treatment does not induce TG accumulation in hepatocytes by altering fatty acid metabolism.

**Fig. 2.**
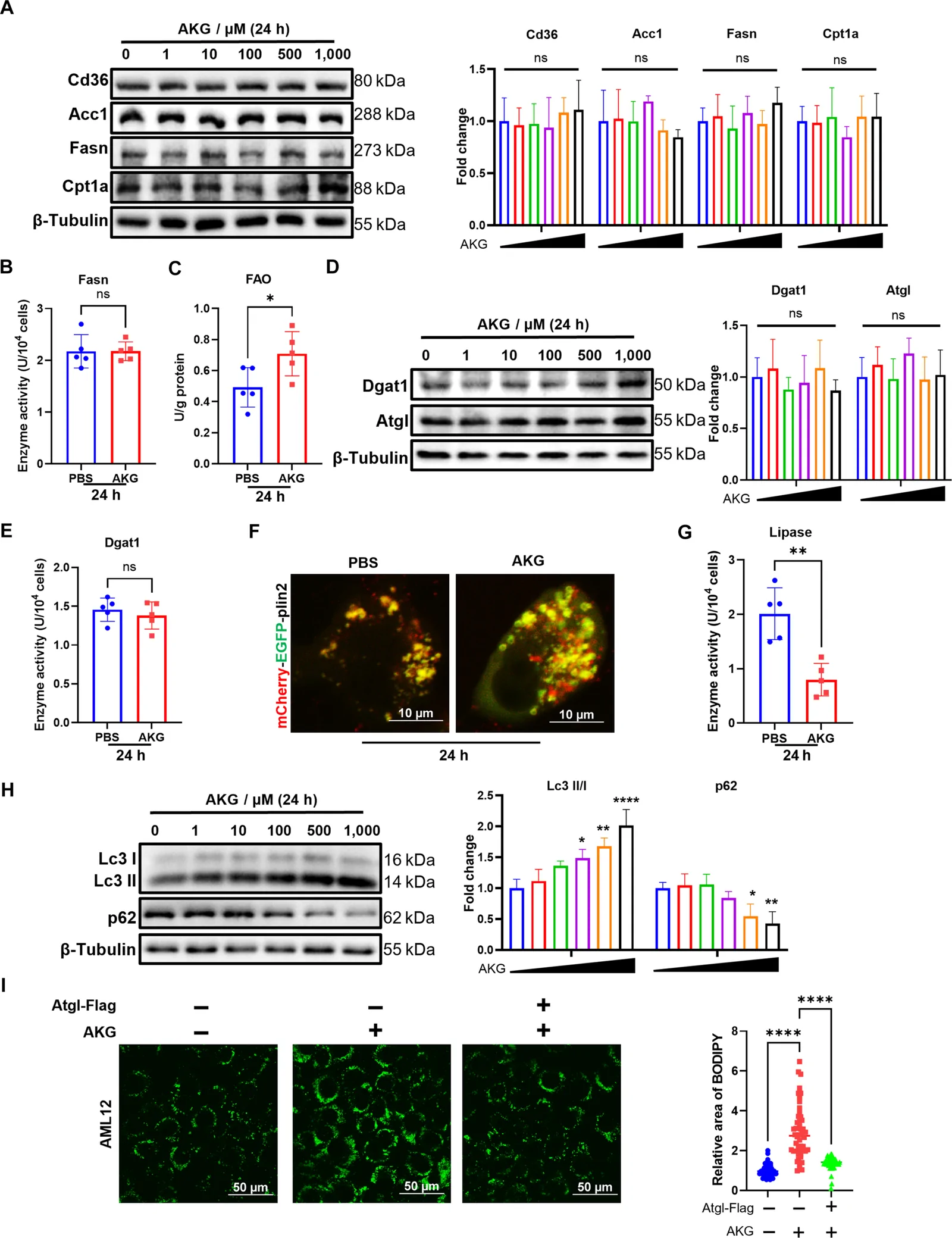
AKG induces lipid accumulation by inhibiting adipose triglyceride lipase (Atgl) activity rather than promoting TG synthesis. (A) Western blot (WB) analysis of the protein expression levels of Cd36, Acc1, Fasn, and Cpt1a in AML12 cells after 24 h of AKG treatment (*n* = 3). (B) The enzyme activity of fatty acid synthase (Fasn) in AML12 cells was detected via a commercial assay kit (*n* = 5). PBS, phosphate-buffered saline. (C) Fatty acid β-oxidation (FAO) enzymatic activity in AML12 cells measured via a commercial kit (*n* = 5). (D) WB analysis of Dgat1 and Atgl protein expression in AML12 cells treated with increasing concentrations of AKG (*n* = 3). (E) DGAT1 enzymatic activity in AML12 cells measured using a commercial kit after treatment with 500 μM AKG for 24 h (*n* = 5). (F) Confocal imaging performed 24 h after treatment with 500 μM AKG and 500 nM Torin1 to induce lipophagy, following 24-h overexpression of mCherry-EGFP-Plin2. (G) Lipase activity in AML12 cells measured using a commercial kit at 24 h 500 μM AKG treatment (*n* = 5). (H) WB analysis of LC3 and p62 (*n* = 3). (I) After 24 h of Atgl-Flag overexpression, AML12 cells were treated with 500 μM AKG for an additional 24 h, followed by boron dipyrromethene (BODIPY) staining and confocal imaging (*n* = 50 cells from 5 independent cultures). Data are presented as mean ± standard deviation. *P < 0.05, **P < 0.01, ***P < 0.001, ****P < 0.0001; ns, not significant. *P* values were determined using an unpaired Student *t* test (C, E, and G) and one-way analysis of variance (ANOVA) followed by Bonferroni multiple comparisons test (A, D, H, and I).

We subsequently investigated whether AKG treatment affects TG metabolism. Diacylglycerol acyltransferase 1 (Dgat1) is the key enzyme directly responsible for TG synthesis. AKG treatment did not affect the protein expression of Dgat1 or its enzymatic activity (Fig. 2D and E and Fig. S4D and E). TGs are mobilized through 2 pathways: lipophagy and lipolysis [11]. The lipophagy pathway relies on lysosomal degradation of lipid droplets [12]. Following previously reported methods [13], we found that AKG treatment promoted lipophagy via a lipophagy probe at both 6 and 24 h of treatment (Fig. 2F and Fig. S4F). This phenomenon, seemingly contradictory to the observed TG accumulation, may serve as a compensatory mechanism aimed at alleviating the hepatocytic lipid burden. TG hydrolysis is another key pathway for TG degradation [14]. We examined Atgl, a crucial enzyme in the first step of lipolysis [15], and found that AKG treatment did not affect its protein expression (Fig. 2D and Fig. S4D). Surprisingly, lipase activity was significantly inhibited by AKG at 24 h but not at 6 h (Fig. 2G and Fig. S4G). AKG treatment for 24 h also enhanced autophagic flux in hepatocytes (Fig. 2H). To confirm whether Atgl activity was responsible for the accumulation of TGs, we promoted TG degradation by overexpressing Atgl-Flag (Fig. 2I and Fig. S7A). The results showed that Atgl-Flag overexpression significantly reduced AKG-induced lipid accumulation (Fig. 2I and Fig. S7A). In summary, these data suggest that AKG treatment induces lipid accumulation by inhibiting the lipase activity of Atgl.

### AKG treatment increases Cidec protein expression to inhibit Atgl activity and contributes to hepatic steatosis

To clarify the molecular mechanism by which AKG inhibits lipase activity, we performed immunoprecipitation (IP) followed by mass-spectrometry analysis to identify proteins that interact with Atgl. The mass-spectrometry dataset was analyzed in conjunction with the lipid metabolism pathway gene set from the GeneCards database and the “negative regulation of lipid catabolic process” gene set from the Gene Ontology database. A Venn diagram was used to visualize the results, and only Cidec was included in all the datasets (Fig. 3A). Western blot (WB) analysis was used to validate the mass-spectrometry results, revealing an interaction between Cidec and Atgl (Fig. 3B). Immunofluorescence also demonstrated the colocalization of Cidec and Atgl in AML12 cells (Fig. 3C). The interaction between Cidec and Atgl was significantly enhanced in the liver tissues of 24th week AKG-fed mice (Fig. 3D). Importantly, incubation of recombinant Cidec with Atgl significantly decreased the lipase activity of recombinant Atgl (Fig. 3E).

**Fig. 3.**
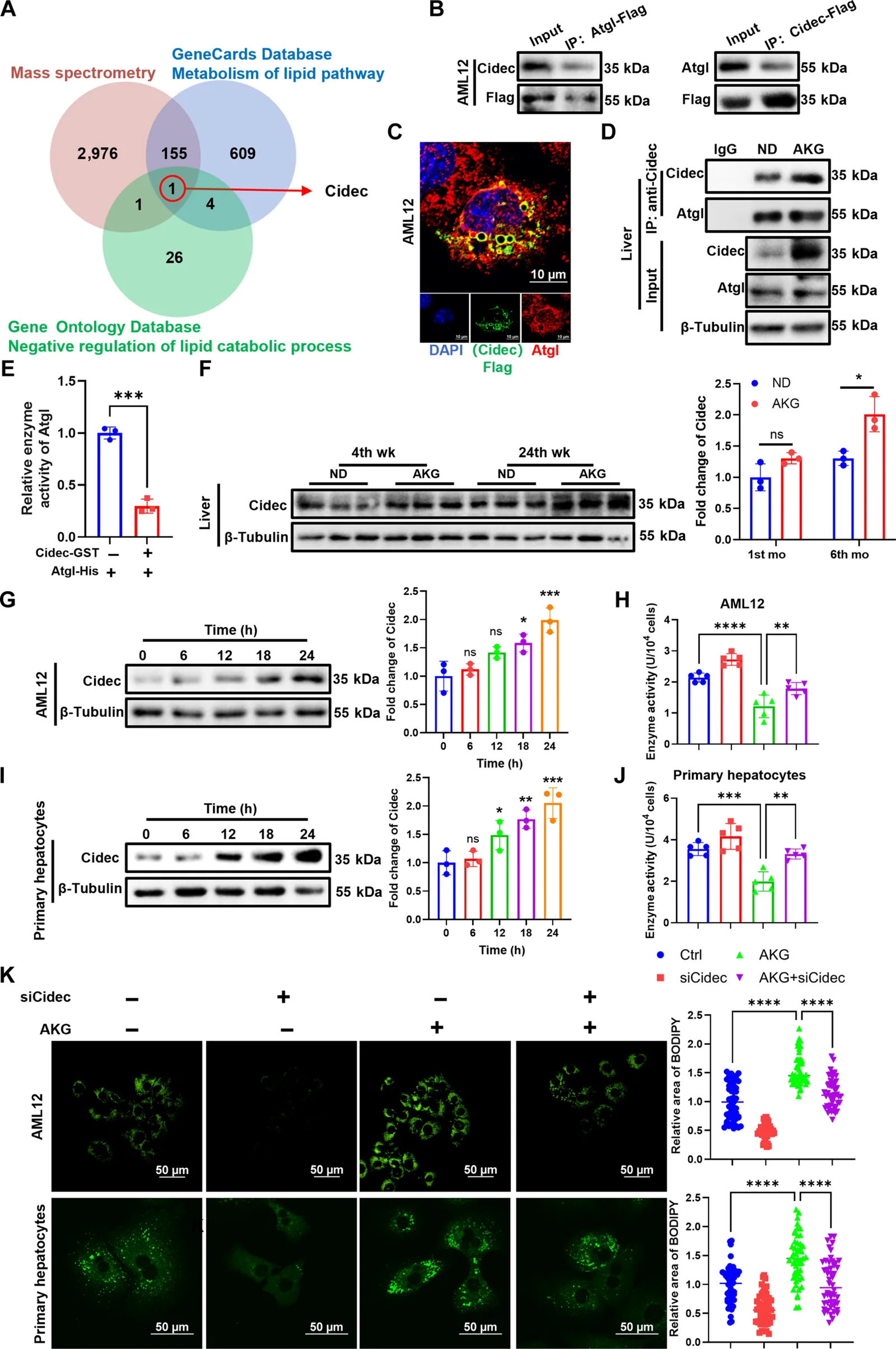
AKG suppresses Atgl lipase activity by promoting cell death-inducing DNA fragmentation factor 45-like effector C (Cidec) protein expression. (A) Co-immunoprecipitation (Co-IP) was performed in AML12 cells to pull down Atgl-Flag, followed by mass-spectrometry analysis of the interacting proteins. The mass-spectrometry results were analyzed in conjunction with the *Metabolism of lipids* pathway dataset from the GeneCards database and the *Negative regulation of lipid catabolic process* dataset from the Gene Ontology (GO) database, and the overlap was visualized using a Venn diagram. (B) Immunoprecipitation (IP) was conducted in AML12 cells to pull down Cidec-Flag or Atgl-Flag, and WB analysis was used to detect the interaction between Atgl and Cidec. DAPI, 4′,6-diamidino-2-phenylindole. (C) Immunofluorescence staining was used to visualize the colocalization of Atgl (green) and Cidec-Flag (red) in AML12 cells. IgG, immunoglobulin G. (D) IP was performed to assess the interaction between Cidec and Atgl in liver tissue. (E) Lipase activity of recombinant ATGL was measured using a commercial kit, with or without pre-incubation with recombinant Cidec (*n* = 3). (F) WB analysis of Cidec protein levels in liver tissues (*n* = 6). (G and I) Protein expression of Cidec was assessed by WB in AML12 cells and primary hepatocytes treated with 500 μM AKG (*n* = 3). (H and J) In AML12 cells and primary hepatocytes, Cidec was knocked down using siRNA for 24 h, followed by treatment with 500 μM AKG for another 24 h. Lipase activity was then measured using a commercial assay kit (*n* = 5). (K) In AML12 cells and primary hepatocytes, Cidec was knocked down and cells were treated with AKG. Lipid droplets were labeled with BODIPY and visualized using confocal microscopy (*n* = 50 cells from 5 independent cultures). Data are presented as mean ± standard deviation. *P < 0.05, **P < 0.01, ***P < 0.001, ****P < 0.0001; ns, not significant. *P* values were determined using an unpaired Student *t* test (E and F) and one-way ANOVA followed by Bonferroni multiple comparisons test (G to K).

In mouse liver tissues, the protein expression of Cidec was significantly increased at the 24th week of AKG treatment but not at the 4th week (Fig. 3F). In AML12 cells and primary hepatocytes, AKG treatment increased Cidec protein expression in a time-dependent manner (Fig. 3G and I). More importantly, knockdown of Cidec expression significantly improved the AKG-induced reduction in lipase activity in AML12 cells and primary hepatocytes (Fig. 3H and J). To investigate the effect of Cidec on lipid droplet degradation, BODIPY-labeled lipid droplets were used, and the results revealed that knocking down Cidec significantly suppressed the AKG-induced increase in lipid droplet area in AML12 cells and primary hepatocytes (Fig. 3K and Fig. S7B).

To further clarify the importance of Cidec in AKG-mediated hepatic steatosis, we used an adeno-associated virus serotype 8 (AAV8) vector carrying a short hairpin RNA (shRNA) targeting mouse Cidec mRNA driven by the hepatocyte-specific thyroxine-binding globulin promoter. The virus was administered via tail vein injection to specifically knock down hepatocytic Cidec expression. Because 16 weeks of 2% AKG feeding already led to significantly increased serum TG levels, and because this duration is substantially longer than the 2 weeks used in previous AKG studies [8], we used a 16-week 2% AKG diet for the knockdown rescue experiments. We found that hepatocyte-specific knockdown of Cidec expression significantly reversed the reduction in hepatic lipase activity (Fig. 4A to D). After 16 weeks, knockdown of Cidec expression did not alter body weights but significantly reduced the AKG-induced increase in serum TG levels (Fig. 4E and F). In addition, Cidec expression knockdown markedly inhibited the AKG-mediated increases in hepatic TG contents, liver weights, and liver-to-body weight ratios (Fig. 4G to I). Levels of the hepatic injury marker AST and ALT were also significantly reduced after Cidec expression was knocked down (Fig. 4J and K). Oil Red O staining of liver tissues revealed that AAV8-mediated Cidec expression knockdown significantly attenuated AKG-induced hepatic steatosis (Fig. 4L). Taken together, these results suggest that AKG induces hepatic steatosis by increasing the protein levels of Cidec in liver cells.

**Fig. 4.**
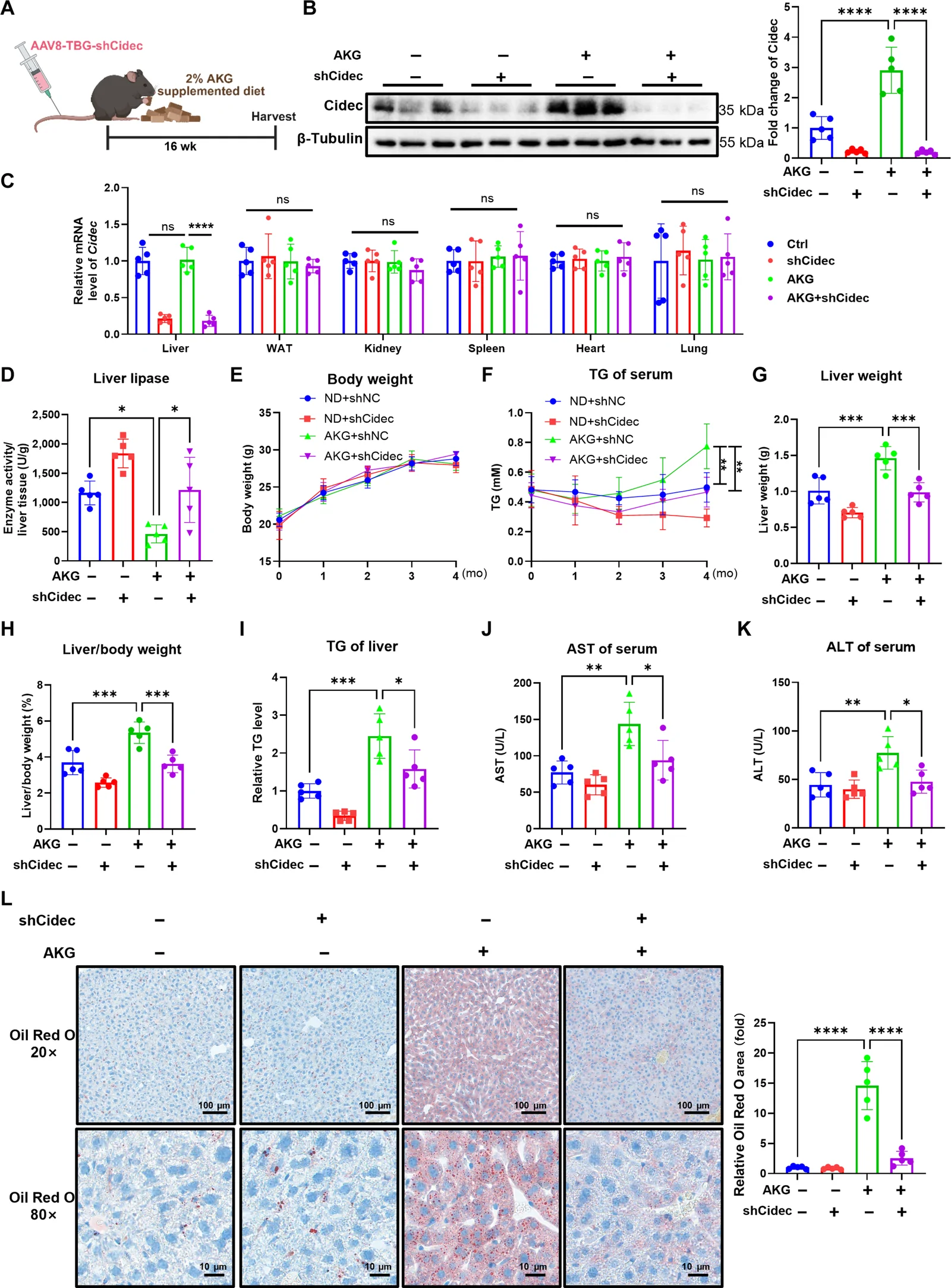
Knockdown of Cidec via adeno-associated virus serotype 8 (AAV8) significantly inhibited AKG-induced hepatic lipid accumulation. (A) Injection of AAV8 and 2% AKG feeding animal model. TBG, thyroxine binding globulin. (B) Cidec protein levels in the liver were measured after 16 weeks (*n* = 5). (C) Quantitative polymerase chain reaction (qPCR) analysis of *Cidec* mRNA levels in various tissues (*n* = 3). (D) The lipase activity of liver was measured after 16 weeks (*n* = 5). (E) Body weight was monitored monthly (*n* = 5). (F) Serum TG levels were measured monthly (*n* = 5). (G) The weight of the liver (*n* = 5). (H) The percentage of liver weight relative to total body weight (*n* = 5). (I) The levels of TG in the liver (*n* = 5). (J) Serum AST levels were measured monthly (*n* = 5). (K) Serum ALT levels were measured monthly (*n* = 5). (L) Oil Red O staining of liver tissue sections (*n* = 5). Data are presented as mean ± standard deviation. *P < 0.05, **P < 0.01, ***P < 0.001, ****P < 0.0001; ns, not significant. *P* values were determined using one-way ANOVA followed by Bonferroni multiple comparisons test (B to L).

### Trim21-dependent down-regulation of ubiquitination enhances Cidec protein stability in AKG-treated hepatocytes

Since AKG treatment did not alter the mRNA expression of *Cidec* in either the liver or hepatocytes (Fig. 5A and B), we propose that AKG increases the protein expression of Cidec by affecting its half-life. Treatment with cycloheximide, which blocks all protein synthesis, revealed that AKG significantly extended the half-life of Cidec (Fig. 5C). To determine the Cidec degradation pathway, we used MG-132 and chloroquine to inhibit the ubiquitin–proteasome pathway and the lysosomal pathway (Fig. 5D). WB analysis revealed that inhibiting the ubiquitin–proteasome pathway significantly increased the protein expression of Cidec (Fig. 5D). Additionally, IP of Cidec-Flag revealed that AKG treatment reduced the ubiquitination level of Cidec-Flag (Fig. 5E).

**Fig. 5.**
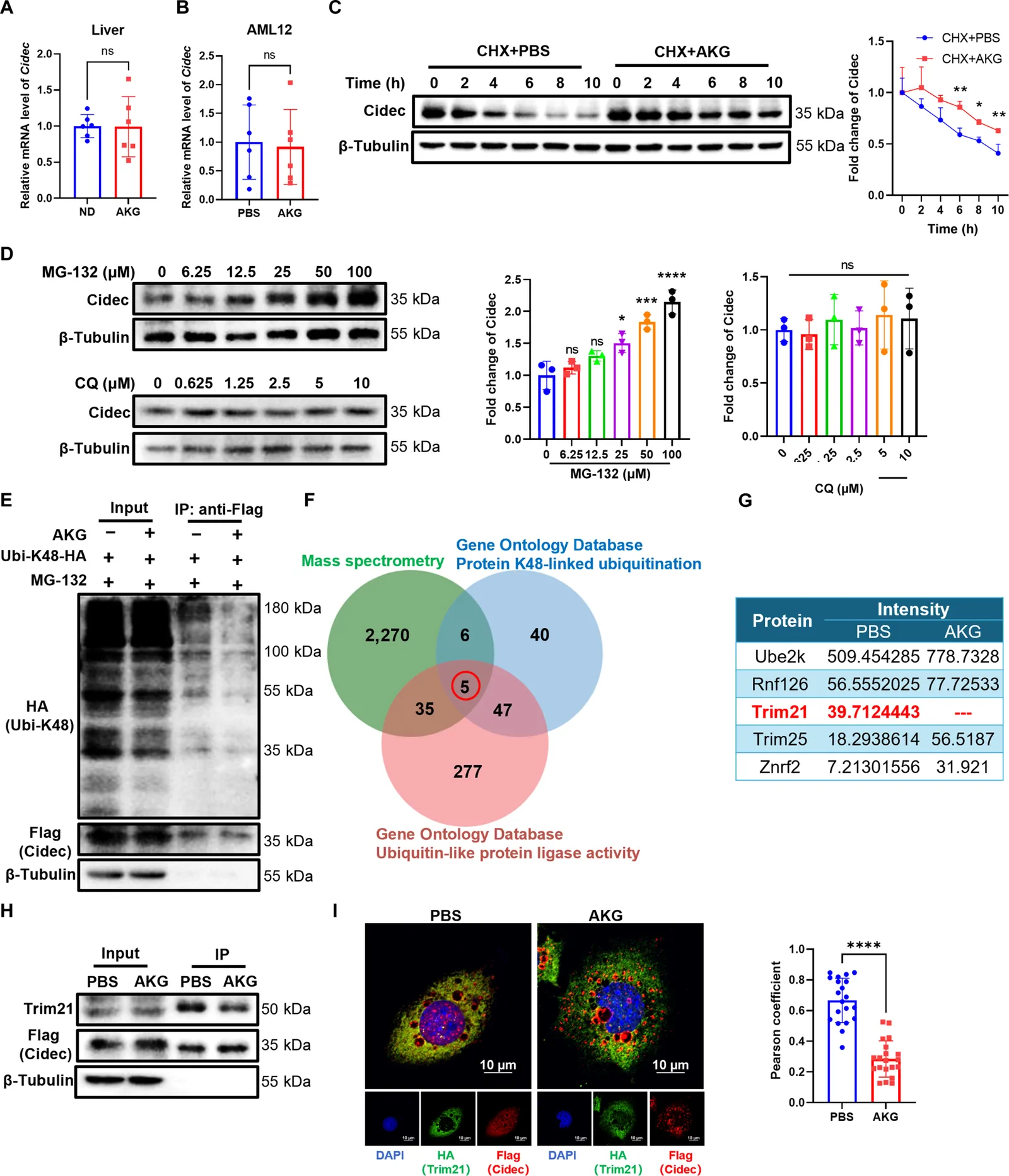
AKG enhances the stability of Cidec by reducing its interaction with tripartite-motif protein 21 (Trim21). (A) qPCR was performed to determine the mRNA expression levels of *Cidec* in mouse livers (*n* = 7). (B) qPCR was used to assess mRNA expression levels of *Cidec* in AML12 cells (*n* = 6). (C) AML12 cells were treated with 50 μM cycloheximide (CHX) and 500 μM AKG, and the protein expression levels of Cidec were evaluated at various time points via WB (*n* = 3). (D) AML12 cells were treated with concentration gradients of proteasome inhibitor (MG-132) or lysosomal inhibitor (chloroquine, CQ) for 24 h, followed by WB analysis to detect intracellular Cidec protein levels (*n* = 3). (E) AML12 cells were transfected with overexpression plasmids for Ubi-K48-HA and Cidec-Flag. After 24 h, 500 μM AKG was added, and K48-linked ubiquitination levels of Cidec were assessed via IP–WB 24 h posttreatment. (F) Co-IP was performed to pull down Cidec-Flag, followed by mass-spectrometry analysis. The mass-spectrometry results were co-analyzed with GO datasets for “Protein K48-linked ubiquitination” and “Ubiquitin-like protein ligase activity” and presented in the form of a Venn diagram. (G) Intersection genes of the 3 datasets. (H) IP–WB was performed to validate the interaction between Cidec-Flag and Trim21. (I) Immunofluorescence was conducted in AML12 cells to assess the colocalization of Trim21-HA (green) and Cidec-Flag (red) (*n* = 20 cells from 3 independent cultures). Data are presented as mean ± standard deviation. *P < 0.05, **P < 0.01, ***P < 0.001, ****P < 0.0001; ns, not significant. *P* values were determined using an unpaired Student *t* test (A, B, C, and I) and one-way ANOVA followed by Bonferroni multiple comparisons test (D).

The E3 ligase catalyzes the ubiquitination of target proteins via the addition of K48-linked ubiquitin chains, thereby achieving precise degradation of the target protein [16]. To determine whether AKG inhibits the ubiquitination of Cidec by preventing the interaction between the E3 ligase and Cidec, we employed IP–mass spectrometry to identify 2,316 proteins that interact with Cidec in AML12 cells (Fig. 5F). We then analyzed the 2,316 proteins identified in conjunction with proteins associated with protein K48-linked ubiquitination and ubiquitin-like protein ligase activity from the Gene Ontology database. A Venn diagram was used to visualize the shared proteins (Fig. 5F). Among these, the E3 ligase Trim21 was found to interact with Cidec in AML12 cells, whereas its interaction with Cidec was decreased upon AKG treatment (Fig. 5G).

Further validation using IP followed by WB confirmed the mass-spectrometry results (Fig. 5H). Immunofluorescence analysis also revealed that Trim21 and Cidec interact under normal conditions, but their interaction is significantly reduced after AKG treatment (Fig. 5I).

Knocking down Trim21 expression via small interfering RNA (siRNA) significantly reduced the K48 ubiquitination level of Cidec and increased the protein expression of Cidec (Fig. S5A and B). Lipase activity was also significantly reduced following Trim21 expression knockdown (Fig. S5C). The area of BODIPY-labeled lipid droplets significantly increased after Trim21 knockdown (Fig. S5D). Consistent with the above findings, overexpression of Trim21 significantly increased the K48 ubiquitination level of Cidec and inhibited the AKG-induced increase in Cidec protein expression (Fig. S5E and F). Moreover, Trim21 overexpression significantly increased lipase activity and suppressed AKG-induced lipid droplet accumulation (Fig. S5G and H). Mass-spectrometry analysis revealed that potential ubiquitination of Cidec may occur at K75 and K186 (Fig. S6).

Taken together, these findings indicate that AKG inhibits Trim21-mediated ubiquitination and degradation of Cidec by reducing the interaction between Trim21 and Cidec. However, how AKG affects the interaction between Trim21 and Cidec remains unclear.

### “Lactylation–ubiquitination crosstalk” enhances the protein stability of Cidec in AKG-treated hepatocytes

Numerous studies have shown that protein–protein interactions can be influenced by posttranslational modifications (PTMs) [17]. To determine whether AKG affects the ubiquitination of Cidec via other PTMs, we first examined the lactylation, acetylation, and succinylation levels of total protein. The levels of all 3 PTMs increased in a dose-dependent manner after AKG treatment (Fig. 6A). Furthermore, after using IP to purify Cidec protein, the levels of the 3 PTMs were assessed. The results revealed that only the lactylation of Cidec significantly increased after AKG treatment (Fig. 6B and Fig. S7C).

**Fig. 6.**
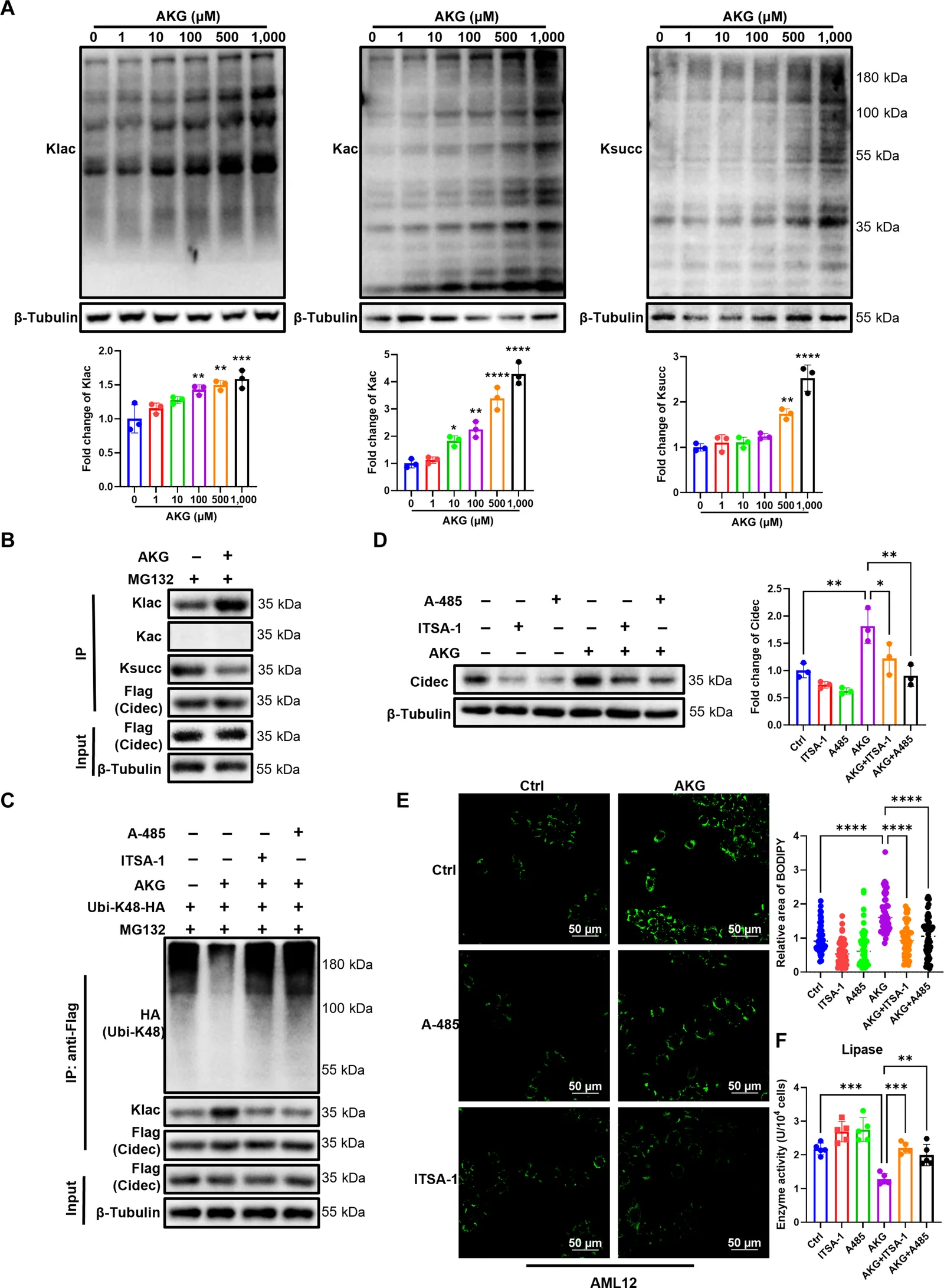
“Lactylation–ubiquitination crosstalk” enhances the stability of Cidec. (A) WB analysis was used to detect the levels of lactylation, acetylation, and succinylation of total proteins (*n* = 3). (B) Detection of lactylation, acetylation, and succinylation levels of Cidec in AML12 cells via IP–WB. (C) IP–WB analysis was used to detect Trim21, lactylation, and ubiquitination levels of Cidec after treatment with AKG, ITSA-1, or A-485. (D) WB analysis of Cidec protein expression in AML12 cells following treatment with 500 μM AKG, 50 μM histone deacetylase agonist (ITSA), or 500 nM P300 inhibitor (A-485) (*n* = 3). (E) BODIPY staining followed by lipid droplet imaging in AML12 cells after AKG, ITSA-1, or A-485 treatment (*n* = 50 cells from 5 independent cultures). (F) Lipase activity was then measured using a commercial assay kit (*n* = 5). Data are presented as mean ± standard deviation. *P < 0.05, **P < 0.01, ***P < 0.001, ****P < 0.0001; ns, not significant. *P* values were determined using one-way ANOVA followed by Bonferroni multiple comparisons test (A, B, C, and E).

P300 and histone deacetylase serve as key writers and erasers for the regulation of protein lactylation [18]. The P300 inhibitor A-485 and histone deacetylase activator ITSA-1 effectively reduced the lactylation level of Cidec while increasing its interaction with Trim21 and the ubiquitination level of Cidec (Fig. 6C and Fig. S7D). Furthermore, treatment with A-485 and ITSA-1 attenuated the AKG-induced increase in Cidec protein expression (Fig. 6D) and reduced the AKG-mediated increase in the area of BODIPY-labeled lipid droplets (Fig. 6E).

Lactate, a critical substrate for lactylation, can increase intracellular lactylation levels [19]. Interestingly, AKG treatment significantly increased lactate levels in liver and AML12 cells (Figs. S7E and S8A). Like AKG treatment, sodium lactate supplementation increased the lactylation level of Cidec but decreased its interaction with Trim21 and the ubiquitination level of Cidec (Fig. S8B). Sodium lactate treatment increased the protein expression of Cidec (Fig. S8C), but it did not affect the mRNA expression of Cidec (Fig. S8D). Moreover, sodium lactate treatment inhibited lipase activity and increased the area of BODIPY-labeled lipid droplets in AML12 cells (Fig. S8E and F).

Lactate dehydrogenase A (Ldha) catalyzes the conversion of pyruvate to lactate [20]. Knockdown of Ldha expression reduced the lactate level and lactylation level of Cidec, increased its interaction with Trim21, and decreased its ubiquitination level (Fig. S8G to I). Although AKG treatment did not increase the protein expression of Ldha, the knockdown of Ldha expression via siRNA significantly inhibited the AKG-induced increase in Cidec expression (Fig. S8G). Furthermore, Ldha expression knockdown enhanced the reduction in lipase activity mediated by AKG (Fig. S8J) and reduced the AKG-induced increase in lipid droplet area (Fig. S8K).

We further employed mass spectrometry to analyze the specific lactylation modification sites on Cidec in hepatocytes following AKG treatment. The results identified lactylation at lysine 124 (K124) of Cidec after AKG treatment (Fig. S9A). To mimic the lactylated and non-lactylated states, we mutated the lysine at position 124 of Cidec to glutamine (Q) and arginine (R), generating the K124Q and K124R mutants, respectively. Compared to wild-type Cidec, the K124Q mutant exhibited weaker interaction with Trim21 and a lower level of ubiquitination (Fig. S9B). The interaction between the K124R mutant and Trim21, as well as its ubiquitination level, was not affected by AKG treatment (Fig. S9C). Overexpression of the K124Q mutant led to lower lipase activity and larger lipid droplet area compared to overexpression of wild-type Cidec (Fig. S9D and E). In the presence of AKG, overexpression of the K124R mutant resulted in higher lipase activity and smaller lipid droplet area relative to overexpression of wild-type Cidec (Fig. S9D and E).

### Pck1 promotes lactate production in AKG-treated hepatocytes

To elucidate the molecular mechanism by which AKG increases lactate levels in hepatocytes, we performed transcriptomic analysis (GSE304106) on liver samples from mice after 24 weeks of AKG consumption. Principal component analysis revealed significant differences in hepatic gene expression following AKG administration (Fig. S10A). Gene Ontology enrichment analysis of the differentially expressed genes between the 2 groups revealed that biological processes related to fatty acid metabolism processes and lipid catabolic processes were enriched, and lipid droplets were significantly enriched in differentially expressed genes related to cellular components (Fig. S10B). These results further confirm that long-term AKG consumption is associated with dysregulated liver lipid metabolism.

Because AKG and lactate are metabolic intermediates in the aerobic and anaerobic glucose oxidation pathways, respectively, we visualized the expression of key enzymes involved in aerobic glycolysis, anaerobic glycolysis, and gluconeogenesis using a heatmap (Fig. 7A). Interestingly, the gene expression of *Pck1*, the first rate-limiting enzyme in gluconeogenesis [21], was significantly up-regulated (Fig. 7A). Quantitative polymerase chain reaction (qPCR) was conducted to validate the transcriptomic sequencing results, which were then mapped onto a glucose metabolism pathway diagram (Fig. 7B). qPCR analysis also revealed that long-term AKG consumption significantly increased *Pck1* gene expression in the mouse liver (Fig. 7B). At the protein level, AKG treatment markedly increased Pck1 expression both in vivo and in vitro (Fig. 7C and D).

**Fig. 7.**
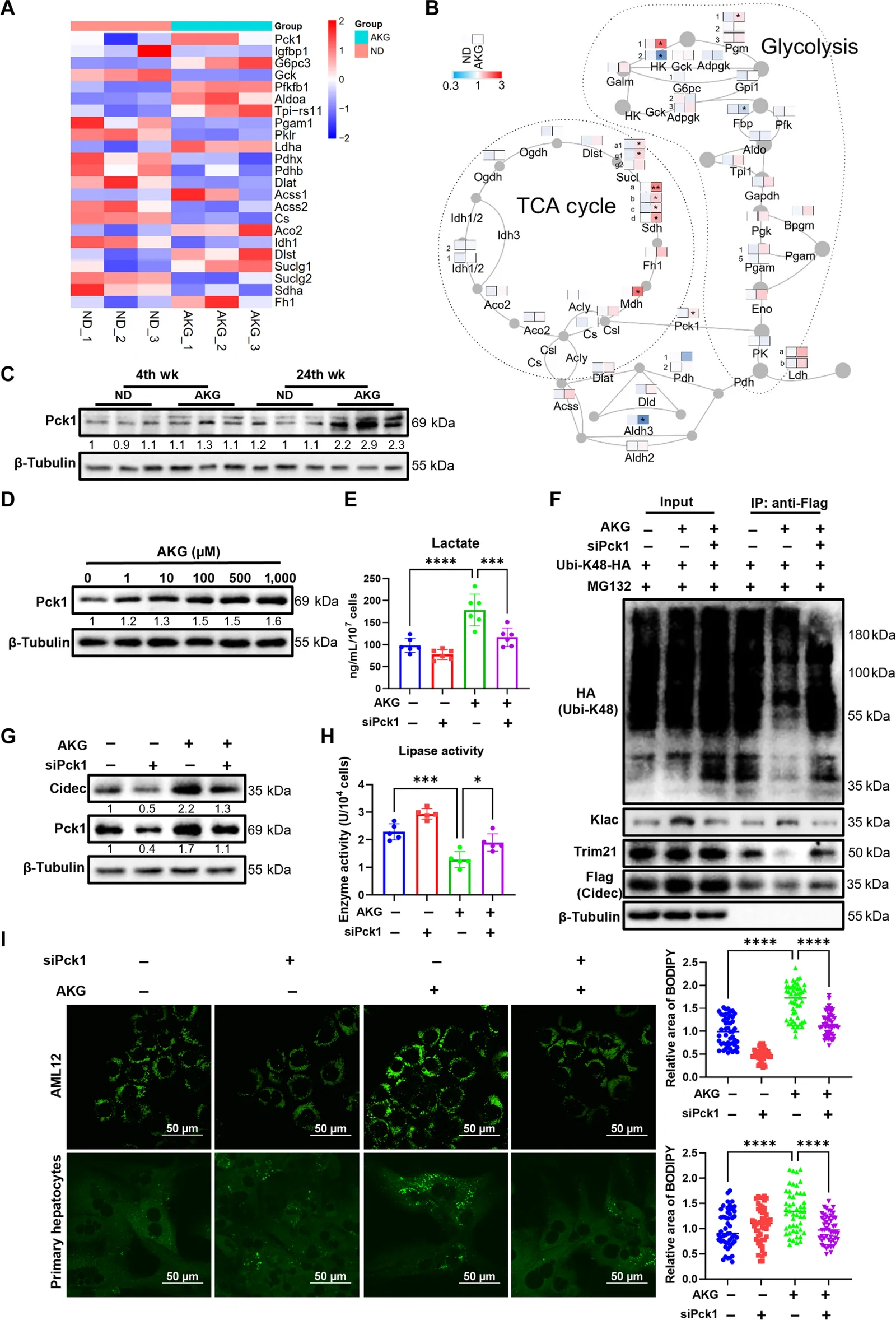
AKG induces lipid accumulation in hepatocytes by promoting PCK1 protein expression. (A) Heatmap shows mRNA expression levels of enzymes involved in glycolysis, gluconeogenesis, and the tricarboxylic acid (TCA) cycle from transcriptomic datasets. (B) qPCR was performed to assess mRNA expression of enzymes related to glycolysis, gluconeogenesis, and the TCA cycle in liver tissues (*n* = 4). (C) WB was used to detect the protein level of Pck1 in mouse liver. (D) WB was conducted to evaluate Pck1 protein expression in AML12 cells. (E) Ultra-performance liquid chromatography–tandem mass spectrometry was used to determine lactate in AML12 cells (*n* = 6). (F) IP–WB analysis was performed to detect Trim21, lactylation, and ubiquitination levels of Cidec. HA, human influenza hemagglutinin. (G) WB was performed to examine protein expression of Cidec and Pck1 in AML12 cells following knockdown of Pck1 combined with AKG supplementation (*n* = 3). (H) Commercial assay kits were used to measure changes in lipase activity in AML12 cells (*n* = 5). (I) BODIPY staining and imaging were performed to visualize lipid droplets in AML12 cells and primary hepatocytes (*n* = 50 cells from 5 independent cultures). Data are presented as mean ± standard deviation. *P < 0.05, **P < 0.01, ***P < 0.001, ****P < 0.0001; ns, not significant. *P* values were determined using an unpaired Student *t* test (B) and one-way ANOVA followed by Bonferroni multiple comparisons test (E, H, and I).

Pck1 catalyzes the conversion of oxaloacetate to phosphoenolpyruvate, which contains a high-energy enol phosphate bond and is highly unstable [22]. Phosphoenolpyruvate is irreversibly converted to pyruvate by pyruvate kinase [23], and pyruvate is the direct precursor of lactate [24]. AKG treatment significantly increased pyruvate levels in AML12 cells (Fig. S10C). Pck1 expression knockdown using siRNA significantly inhibited AKG-induced pyruvate and lactate accumulation in hepatocytes, reduced the level of Cidec lactylation, and increased the level of Cidec ubiquitination (Fig. 7E and F and Fig. S10C). Pck1 expression knockdown using siRNA effectively suppressed the AKG-induced increase in Cidec protein expression (Fig. 7G) and restored lipase activity (Fig. 7H). Moreover, in both AML12 cells and primary hepatocytes, Pck1 expression knockdown significantly inhibited the AKG-induced increase in lipid droplet area (Fig. 7I). In AKG-treated AML12 cells with Pck1 knockdown, further lactate supplementation restored Cidec lactylation, reduced both the interaction between Trim21 and Cidec and Cidec ubiquitination levels, and increased both Cidec protein levels and lipid droplet numbers (Fig. S10D to G).

To further clarify the critical role of Pck1 in the pro-steatotic effects of AKG, a tail vein injection of an AAV8 vector carrying a thyroxine-binding globulin promoter-driven shRNA targeting mouse Pck1 was used to treat mice with AKG-induced hepatic steatosis. The shRNA not only reduced hepatic Pck1 protein expression but also attenuated the AKG-induced increase in Cidec expression (Fig. 8A to C). Lipase activity in liver tissue was significantly increased following Pck1 expression knockdown (Fig. 8D). We monitored the body weights and serum TG levels of the mice monthly (Fig. 8E and F). Pck1 expression knockdown significantly suppressed the AKG-induced increase in serum TG concentration (Fig. 8F). In addition, Pck1 expression knockdown markedly reduced AKG-induced increases in liver weights, liver-to-body weight ratios, hepatic TG contents, and serum AST and ALT levels (Fig. 8G to K). Considering the important role of Pck1 in glycogenesis, we measured liver glycogen levels. Interestingly, while the knockdown of Pck1 expression decreased liver glycogen levels, AKG did not affect liver glycogen levels (Fig. 8L). The low expression of Fbp1 in the liver after AKG consumption may partly explain this phenomenon (Fig. 7B). Consistent with the biochemical data, Oil Red O staining revealed that Pck1 expression knockdown significantly ameliorated AKG-induced hepatic steatosis (Fig. 8M).

**Fig. 8.**
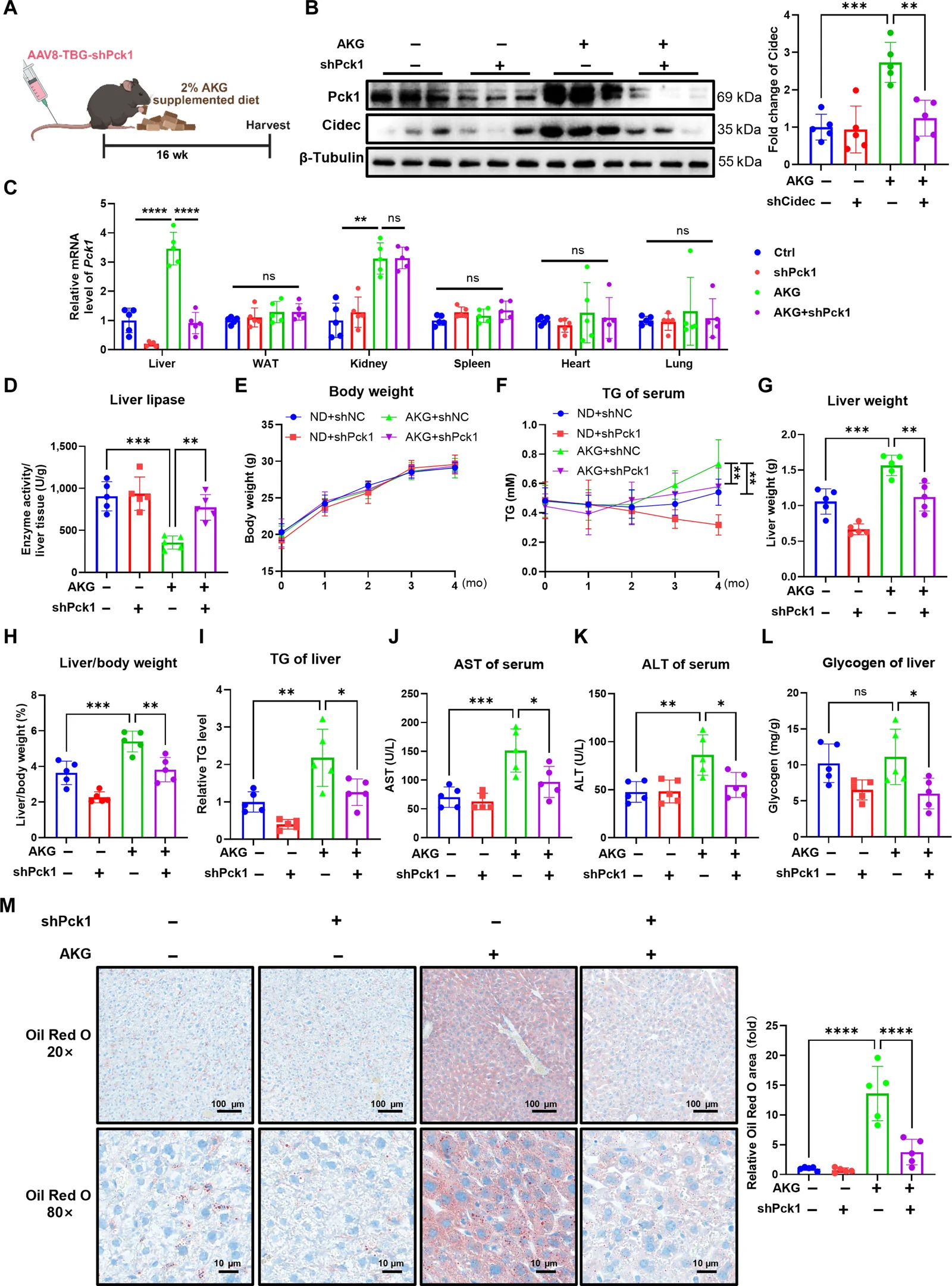
Knockdown of Pck1 by AAV8 significantly inhibited AKG-induced hepatic steatosis. (A) Injection of AAV8 and 2% AKG feeding animal model. (B) Cidec and Pck1 protein levels in the liver were measured after 16 weeks (*n* = 5). (C) qPCR analysis of *Pck1* mRNA levels in various tissues (*n* = 3). (D) The lipase activity of liver were measured after 16 weeks (*n* = 5). (E) Body weight was monitored monthly (*n* = 5). (F) Serum TG levels were measured monthly (*n* = 5). (G) The weight of the liver (*n* = 5). (H) The percentage of liver weight relative to total body weight (*n* = 5). (I) The levels of TG in the liver (*n* = 5). (J) Serum AST levels were measured monthly (*n* = 5). (K) Serum ALT levels were measured monthly (*n* = 5). (L) The levels of glycogen in the liver (*n* = 5). (M) Oil Red O staining of liver tissue sections (*n* = 5). Data are presented as mean ± standard deviation. *P < 0.05, **P < 0.01, ***P < 0.001, ****P < 0.0001; ns, not significant. *P* values were determined using one-way ANOVA followed by Bonferroni multiple comparisons test (B to M).

The Jumonji C-domain containing histone demethylases (JMJs) family regulates methylation levels at histone sites including H3K4, H3K9, H3K27, and H3K36 [25]. As a cofactor of JMJs, AKG can activate gene expression by modulating histone methylation at these sites [26]. To investigate the specific molecular mechanism by which AKG activates *Pck1* transcription, we analyzed the enrichment of H3K4me3, H3K9me3, H3K27me3, and H3K36me3 in the *Pck1* promoter region using the Cistrome DB database (Fig. S11A). The results revealed significant enrichment of H3K4me3 and H3K36me3 in the *Pck1* promoter (Fig. S11A). In hepatocytes, AKG reduced H3K4me3 levels in a dose-dependent manner without affecting trimethylation at the other 3 sites (Fig. S11B). More importantly, chromatin immunoprecipitation–qPCR confirmed that AKG significantly decreased H3K4me3 enrichment at the *Pck1* promoter, and that subsequent addition of CPI-455 restored this enrichment (Fig. S11C). The histone demethylase 5 (Kdm5), a member of the JMJs family, specifically regulates H3K4 methylation [27]. The Kdm5 inhibitor CPI-455 markedly attenuated the AKG-mediated reduction of H3K4me3 and restored the AKG-induced down-regulation of Pck1 and Cidec protein levels (Fig. S11D). Furthermore, CPI-455 reversed the AKG-induced decrease in lipase activity and reduced lipid droplet accumulation in hepatocytes (Fig. S11E and F). Among the Kdm5 family members, Kdm5b has been reported to participate in regulating TG metabolism in hepatocytes [28]. To further define the mediator through which AKG acts, we knocked down Kdm5b to assess its role. The results demonstrated that Kdm5b knockdown, similar to CPI-455 treatment, effectively reduced AKG-induced Pck1 and Cidec expression, increased lipase activity, and decreased AKG-induced lipid accumulation in hepatocytes (Fig. S11G to I).

In summary, these data indicate that AKG promotes *Pck1* transcription at least in part through Kdm5b-H3K4me3, leading to increased lactate production, which subsequently promotes Cidec lactylation and ultimately contributes to hepatic steatosis.

## Discussion

In this study, we found that long-term AKG intake as a dietary supplement could contribute to hepatic steatosis in mice. Mechanistically, our findings suggest that AKG increases pyruvate and lactate by up-regulating Pck1 expression, leading to increased lactylation of Cidec. Through “lactylation–ubiquitination crosstalk”, AKG inhibits Trim21-mediated ubiquitination and degradation of Cidec, thereby increasing the stability of the Cidec protein. Consequently, elevated Cidec expression further suppresses Atgl lipase activity, reduces TG hydrolysis, and results in hepatic steatosis (Fig. S12).

In recent studies, AKG has been regarded as a dietary supplement because of its antidiabetic and anti-aging properties [29]. However, some studies have caused controversy regarding its metabolic regulatory effects [30,31]. Previous studies have focused on the molecular mechanisms of AKG in conditions such as hyperlipidemia, obesity, and diabetes [6–8]. Supplementation with AKG for 9 d reduces liver TG levels in hyperlipidemic mice but has no effect on normal mice [7]. Supplementing with AKG for 1 week reduces blood glucose levels in diabetic mice but does not affect blood glucose levels in nondiabetic mice [6]. Supplementing with AKG for 2 weeks reduces body weight in obese mice but has no effect on the body weights of nonobese mice [8]. These findings suggest that AKG may have different mechanisms under physiological and pathological conditions.

To explore the mechanism by which AKG induces hepatic steatosis, we examined the effects of AKG on fatty acid and TG metabolism. Surprisingly, lipophagy, one of the main pathways of TG degradation [12], was not inhibited by AKG but rather was enhanced. We hypothesize that lipophagy, an alternative degradation pathway that is independent of TG hydrolases, may be enhanced as a compensatory mechanism. Commercial assay kits revealed that the enzymatic activity of FAO was increased in hepatocytes following AKG treatment, which is similar to the findings of Liu et al. [32]. Research by Meçe et al. [33] has demonstrated that fatty acids generated through lipophagy can serve as essential substrates for FAO. Therefore, we propose that the fatty acids produced by lipophagy are consumed by FAO in AKG-treated hepatocytes. As a key enzyme in the TG hydrolysis pathway, Atgl plays a crucial role in the development of hepatic steatosis [14]. Therefore, we propose that the reduction in Atgl lipase activity is a critical mechanism by which AKG induces hepatic steatosis.

In 2014, Grahn et al. [34] discovered that Cidec directly interacts with Atgl and inhibits its TG hydrolase activity in adipocytes. Here, we extend this regulatory paradigm to hepatocytes, demonstrating that Cidec similarly binds Atgl and suppresses its enzymatic activity in liver cells. Our study further reveals that AKG-induced lactylation stabilizes Cidec and promotes its accumulation, thereby identifying a novel posttranslational mechanism that elevates Cidec protein levels under nutrient stress and adds a new regulatory layer to hepatic steatosis.

Although *Cidec* is an evolutionarily conserved gene overall, the specific lysine residue K124 that we identified as a lactylation site in mouse *Cidec* is not conserved in human *CIDEC*. The corresponding position in the human protein is occupied by a glutamine residue rather than a lysine, and the surrounding amino acid sequence also differs considerably between the 2 species. This observation is consistent with emerging evidence that, while lactylation as a modification is functionally conserved across species, the specific modified sites can diverge during evolution. Global profiling studies have shown that only a limited subset of lactylation sites are conserved between humans and rodents, with many sites exhibiting species-specific patterns [35]. This site-level divergence is particularly relevant for translational interpretation, as it implies that the lactylation-dependent regulation of Cidec stability that we describe in mice may not be directly extrapolatable to human CIDEC. Although K124 is not conserved in human CIDEC (human Q124), lactylation may still regulate CIDEC stability through functionally conserved mechanisms. For instance, hypoxia-inducible factor 1-alpha lactylation occurs at distinct residues in mice and humans but similarly enhances protein stability by blocking von Hippel–Lindau-mediated degradation [36]. Therefore, the specific lactylation sites and functional conservation of human CIDEC warrant further investigation.

As an intermediate product of the TCA cycle, AKG can be converted into succinyl-CoA, which serves as a substrate for succinylation [4,37]. Moreover, through the malate cycle and Pck1, AKG can exit the mitochondria and be converted into pyruvate and lactate. Pyruvate can be further processed into the acetylation substrate acetyl-CoA [38], whereas lactate can be converted into the glycosylation substrate lactyl-CoA [39]. Therefore, we examined the levels of succinylation, acetylation, and lactylation in hepatocytes following AKG treatment. While our findings demonstrate that AKG elevates lactate levels, direct evidence from ^13^C-labeled metabolic flux analysis is warranted in future investigations.

PTMs play critical roles in regulating the biological functions of proteins [40], and different PTMs can influence each other [41]. Several recent studies have demonstrated that protein lactylation can antagonize ubiquitination: Zhang et al. [42] reported that PKM2 lactylation inhibits its degradation; Li et al. [43] showed that lactylation of the E3 ligase NEDD4 impairs its ability to mediate ubiquitination. These reports establish the general concept that lactylation can regulate protein stability by inhibiting ubiquitination. We show that AKG, through the Pck1–lactate axis, induces Cidec lactylation at K124, which may in turn disrupt Trim21-mediated ubiquitination and degradation of Cidec. This lactylation–ubiquitination crosstalk represents a previously unrecognized mechanism by which AKG reshapes hepatic lipid droplet protein composition and suppresses Atgl activity in hepatocytes. Thus, our findings extend the emerging concept of lactylation as a regulator of protein stability to the context of hepatic lipid metabolism and position the lactylation–ubiquitination axis as the central mechanistic novelty of this work. However, the specific mechanism by which K124 lactylation inhibits Trim21-mediated ubiquitination of Cidec remains unclear. Moreover, because lysine mutations are not specific for lactylation, future studies should use in vitro lactylation assays to directly confirm the specific role of K124 lactylation.

At the transcriptional level, we found that AKG up-regulates Pck1, which is the first rate-limiting enzyme of gluconeogenesis, while down-regulating Fbp1. This finding partly aligns with the work of Yuan et al. [6], who reported that AKG ameliorates diabetes by inhibiting gluconeogenesis. More importantly, we demonstrate that the up-regulation of Pck1 drives an increase in pyruvate and lactate, providing the substrate for Cidec lactylation. Previous studies have separately linked Pck1 to lipid metabolism: Gómez-Valadés et al. [44] showed that Pck1 knockdown reduces TG in diabetic mice, and Xu et al. [45] discovered that Pck1 promotes lipogenesis via phosphorylation of INSIG1/2. Our work significantly extends these fragmented observations by elucidating a novel mechanism: Pck1-mediated lactate production promotes Cidec lactylation, which in turn suppresses Atgl activity.

For a long time, H3K4me3 has been regarded as a hallmark of transcriptional activation [46]. However, Wang et al. and Hu et al. [47,48] independently discovered that H3K4me3 can inhibit transcription by causing RNA polymerase II pausing in 2023. Our data provide independent support for this emerging concept in a novel metabolic context: We found that AKG, at least in part through Kdm5b, reduces H3K4me3 at the Pck1 promoter to activate its transcription. This observation not only reinforces the repressive function of H3K4me3 but also identifies a specific, AKG-sensitive genomic locus where this regulation occurs, linking AKG to histone-demethylation-dependent gene activation. Nevertheless, the precise mechanism by which H3K4me3 represses *Pck1* transcription remains unclear and is not established by our data. Based on previous reports, we speculate that it may involve RNA polymerase II pausing, but this remains a hypothesis to be tested.

Unfortunately, while we have reported the adverse metabolic effects associated with long-term AKG administration, we have not systematically investigated its safety threshold or optimal duration of use. This critical aspect warrants further investigation, particularly through clinical studies to establish evidence-based guidelines for its safe application. Moreover, this study was conducted exclusively in male mice because the cyclical estrogen fluctuations in females profoundly affect lipid metabolism, and using males in this initial mechanistic study reduced hormonal variability. Future work should include females to address sex as a biological variable. Future research could further clarify the effects of AKG on whole-body metabolism by meticulously measuring energy intake and expenditure in individual mice. Finally, the AKG dose used in mice was selected based on previous animal studies, and its direct relevance to human dietary-supplement consumption remains unclear. To date, human pharmacokinetic data on circulating AKG after oral intake are lacking, which prevents reliable extrapolation of our findings to clinical settings. Future clinical studies are needed to define human-relevant exposure and safety profiles.

Our findings indicate that long-term AKG intake contributes to hepatic lipid accumulation in mice. However, these conclusions are restricted to the mouse experimental setting and should not be extrapolated to humans without further validation. Future studies are needed to determine whether similar metabolic effects occur in humans and to define appropriate dosage and duration across different populations. These findings also highlight the importance of considering context-dependent effects when evaluating the beneficial actions of AKG reported previously.

## Materials and Methods

### Animal experiments

Male C57BL/6 mice (8 wk of age, 18 to 23 g) were obtained from Shulaibao Biotechnology Co., Ltd. (Wuhan, China) and housed under specific pathogen-free conditions in a controlled environment (temperature: 22 ± 2 °C; humidity: 55 ± 10%; 12-h light/dark cycle) with unrestricted access to standard chow and water.

Mice were randomly assigned to different experimental groups receiving 2% AKG (K8030; Solarbio, Beijing, China) supplementation via either the diet (*n* = 7 per group) or drinking water (*n* = 7 per group). The pH of the AKG administered to the mice was adjusted to 7.4 using NaOH. Control group mice received standard diet and drinking water. The intervention lasted for 24 weeks. Body weight, blood glucose, and blood lipid profiles were measured at 4-week intervals. At the end of the treatment period, mice were euthanized, and liver tissue and serum samples were collected for downstream analyses. The laboratory technicians were not informed of the mouse group assignments prior to conducting the experiments. All animal procedures were approved by the Committee of Experimental Animal Centre of Servicebio under the ethical approval no. 2022089.

### Adeno-associated virus injection

According to previously described methods [49], 8-week-old male C57BL/6 mice were infected via tail vein injection with AAV8-Cidec-shRNA-enhanced green fluorescent protein (EGFP) or AAV8-Pck1-shRNA-EGFP (Lio Biotechnology Co., Ltd, Hunan, China). AAV8-Control-EGFP was used as the control. Each mouse received 1 × 10^11^ genome copies of the virus. Four weeks after AAV8 injection, mice were administered penicillin and streptomycin.

### AML12 cell culture

AML12 cells were purchased from Wuhan Procell Life Technology Co. Ltd (CL-0602; Procell, Wuhan, China). Cells were cultured in Dulbecco’s modified Eagle medium (DMEM)/Nutrient Mixture F-12 (PM150312; Procell) supplemented with 10% fetal bovine serum and 1% insulin-transferrin-selenium solution (60708ES10; Yeasen, Shanghai, China) under standard conditions (37 °C; 5% CO_2_). Experiments were conducted when cells reached appropriate confluence. For AKG treatment, the pH was adjusted to neutral using NaOH.

### Isolation and culture of primary hepatocytes

Primary hepatocytes were isolated from 8-week-old male C57BL/6J according to the previous method [50].

Mice were anesthetized via intraperitoneal injection of ether. The abdominal skin was incised along a “U”-shaped line using curved scissors, followed by opening the peritoneum to expose the abdominal cavity. A catheter was inserted into the inferior vena cava, and prewarmed perfusion solution I [1 mL HEPES (G4210; Servicebio, Wuhan, China) + 5 mL 130 mM EDTA (HY-Y0682; MCE, NJ, USA)] was infused at a rate of 5 mL/min. The portal vein was cut to allow outflow, and perfusion continued until the liver appeared pale yellow. The perfusate was then switched to collagenase type IV solution (CLS-4, Worthington, NJ, USA) (5 mL/min, total volume: 40 mL). The liver was transferred to a sterile culture dish. After removing the fibrous capsule in DMEM containing 1% penicillin–streptomycin (G4003; Servicebio), hepatocytes were gently released by agitating the tissue in DMEM supplemented with 10% fetal bovine serum and 1% penicillin–streptomycin. The cell suspension was filtered through a 70-μm nylon mesh and centrifuged at 50×*g* for 1 to 2 min at 4 °C. The washing step was repeated 2 to 3 times to remove collagenase, dead cells, and debris. The resulting hepatocytes were then used for downstream experiments. For AKG treatment, the pH was adjusted to neutral using NaOH.

### Statistical analysis

According to previously described methods [51], all statistical analyses were performed using GraphPad Prism (version 9.0, San Diego, CA, USA). Data are presented as mean ± standard deviation. The normality of data distribution was verified using the Shapiro–Wilk test. For comparisons between 2 independent groups, unpaired Student *t* test was applied. For multiple group comparisons, one-way analysis of variance (ANOVA) was employed, followed by Bonferroni multiple comparisons test for planned comparisons against a single control group. The threshold for statistical significance was set at *P* <0.05. Specific statistical tests and sample sizes (*n*) for each experiment are explicitly stated in the corresponding figure legends. Outcome assessments were performed under consistent blinding. Investigators collecting data and performing analyses were blinded to group allocation.

## Data Availability

The data that support the findings of this study are available from the Gene Expression Omnibus (GSE304106).
