# Supplementary material for: α-Ketoglutarate Promotes Hepatic Steatosis by Enhancing Cidec Stability via Lactylation–Ubiquitination Crosstalk
Source: Research (Wash D C). 2026 Nov 9;9:1471. doi: 10.34133/research.1471 (PMC13745763; doi:10.34133/research.1471)
Supplement: Supplementary file 1 [file research.1471.f1.docx]

SUPPLEMENTARY MATERIALS

**Figure S1-S12**

**Figure. S1 Long-term 2% α-Ketoglutarate (AKG) intake through diet promotes hepatic lipid accumulation.**

**A** Body weight was monitored weekly in C57 mice fed a diet supplemented with 2% AKG (n = 7). **B** Serum TG levels, **C** Blood glucose levels were measured monthly (n = 7). **D&E** Glucose tolerance was assessed by intraperitoneal glucose tolerance test (IPGTT) at week 24, and the area under the curve (AUC) was calculated (n=7). **F&G** Insulin sensitivity was assessed by intraperitoneal insulin tolerance test (IPITT) at week 24, and the AUC was calculated (n=7). **H** Serum AKG levels in mice at first or sixth month after AKG feeding (n = 7). **I** The weight of eWAT in mice at first or sixth month after AKG feeding (n = 7). **J** The percentage of eWAT relative to total body weight (n = 7). **K** The weight of the liver in mice (n = 7). **L** The percentage of liver weight relative to total body weight (n = 7). **M** The levels of TG in the liver of mice (n = 7). **N** Serum AST levels were measured monthly (n = 7). **O** Serum ALT levels were measured monthly (n = 7). **P** Oil Red O staining of liver tissue sections (n = 7). Data are presented as mean ± standard deviation. * *p* < 0.05, ***p* < 0.01, ****p* < 0.001, *****p* < 0.0001; ns, not significant. *p* values were determined using an unpaired student's t-test (A–P).

**Figure. S2 Long-term 1% AKG intake through diet promotes hepatic lipid accumulation.**

**A** Body weight was monitored weekly in C57 mice fed a diet supplemented with 0.1% or 1% AKG (n = 7). **B** Serum TG levels, **C** Blood glucose levels were measured monthly (n = 7). **D&E** Glucose tolerance was assessed by IPGTT at week 24, and the area under the curve (AUC) was calculated (n=7). **F&G** Insulin sensitivity was assessed by IPITT at week 24, and the AUC was calculated (n=7). **H** Serum AKG levels in mice at first or sixth month after AKG feeding (n = 7). **I** The weight of eWAT in mice at first or sixth month after AKG feeding (n = 7). **J** The percentage of eWAT relative to total body weight (n = 7). **K** The weight of the liver in mice (n = 7). **L** The percentage of liver weight relative to total body weight (n = 7). **M** The levels of TG in the liver of mice (n = 7). **N** Serum AST levels were measured monthly (n = 7). **O** Serum ALT levels were measured monthly (n = 7). **P** Oil Red O staining of liver tissue sections (n = 7). Data are presented as mean ± standard deviation. * *p* < 0.05, ***p* < 0.01, ****p* < 0.001, *****p* < 0.0001; ns, not significant. *p* values were determined using an unpaired student's t-test (A–P).

**Figure. S3 AKG treatment increased triglyceride levels in hepatocytes and led to hepatocyte injury.**

**A&B** Cell viability was assessed via the CCK-8 assay in AML12 cells and primary hepatocytes treated with different concentrations of AKG (n=10). **C&D** Cell viability in AML12 cells and primary hepatocytes treated with 500 μM AKG for various time points (n=10). **E&F** Biochemical assay to measure TG levels for various time points (n=3). **G&H** Biochemical assay to measure TC levels for various time points (n=3). **I&J** BODIPY staining of AML12 cells and primary hepatocytes treated with 500 μM AKG for 6h or 24h, followed by observation using laser confocal microscopy (n=50 cells from 5 independent cultures). Data are presented as mean ± standard deviation. * *p* < 0.05, ***p* < 0.01, ****p* < 0.001, *****p* < 0.0001; ns, not significant. *p* values were determined using One-way ANOVA followed by Bonferroni multiple comparisons test (A–J).

**Figure. S4 The effect of 6-hour AKG treatment on lipid metabolism-related enzymes in hepatocytes.**

**A** WB analysis of the protein expression levels of Cd36, Acc1, Fasn, Cpt1a in AML12 cells after 6h of AKG treated (n = 3). **B** The enzyme activity of fatty acid synthase (Fasn) in AML12 cells was detected via a commercial assay kit (n=5). **C** Fatty acid β-oxidation (FAO) enzymatic activity in AML12 cells measured using a commercial kit after treatment with 500 μM AKG for 24 hours (n = 5). **D** WB analysis of Dgat1 and Atgl protein expression (n = 3). **E** DGAT1 enzymatic activity in AML12 cells measured using a commercial kit after treatment with 500 μM AKG for 24 hours (n = 5). **F** Confocal imaging performed 3 hours after treatment with 500 μM AKG and 500 nM Torin1 to induce lipophagy, following 24-hour overexpression of mCherry-EGFP-Plin2. **G** Lipase activity in AML12 cells measured using a commercial kit at 6h 500μM AKG treatment (n = 5). Data are presented as mean ± standard deviation. * *p* < 0.05, ***p* < 0.01, ****p* < 0.001, *****p* < 0.0001; ns, not significant. *p* values were determined using an unpaired student's t-test (B, D) and One-way ANOVA followed by Bonferroni multiple comparisons test (A, C).

**Figure. S5 AKG enhances the stability of Cidec by reducing its interaction with Trim21.**

**A** WB analysis of Cidec K48 ubiquitination level after knockdown of Trim21 via siRNA in AML12 cells. **B** WB analysis of the protein levels of Cidec and Trim21 (n=3). **C** Commercial assay kit to measure lipase activity (n=5). **D** Laser confocal microscopy to observe BODIPY-labeled lipid droplets (n=50 cells from 5 independent cultures). **E** WB analysis of Cidec K48 ubiquitination level after overexpression of Trim21. **F** WB analysis of the protein levels of Cidec and Trim21 after 24 hours of AKG treatment following overexpression of Trim21 (n=3). **G** Commercial assay kit to measure lipase activity (n=5). **H** Laser confocal microscopy to observe BODIPY-labeled lipid droplets (n=50 cells from 5 independent cultures). Data are presented as mean ± standard deviation. * *p* < 0.05, ***p* < 0.01, ****p* < 0.001, *****p* < 0.0001; ns, not significant. *p* values were determined using an unpaired student's t-test (B-D) and One-way ANOVA followed by Bonferroni multiple comparisons test (F-H).

**Figure. S6 Ubiquitination sites at K75 and K186 of Cidec**

**A** Representative MS/MS spectra showing potential ubiquitination sites at K75 and K186 of Cidec.

**Figure. S7 AKG promotes the lactylation of Cidec.**

**A** The overexpression of Atgl-Flag in AML12 cells was detected by WB. **B** Knockdown Cidec using siRNA in AML12 cells or primary hepatocytes, then detect the expression level of Cidec by WB (n = 3). **C** WB analysis of Cidec lactylation levels in liver tissue. **D** Reduce the lactylation levels in AML12 cells using ITSA and A-485, then detect them via WB. **E** Lactate concentration in mouse liver was measured (n = 5). Data are presented as mean ± standard deviation. * *p* < 0.05, ***p* < 0.01, ****p* < 0.001, *****p* < 0.0001; ns, not significant. *p* values were determined using a One-way ANOVA followed by Bonferroni multiple comparisons test (B, D, E).

**Figure. S8 The increase in lactate production leads to enhanced lactylation of Cidec in hepatocytes treated with AKG.**

**A** Ultra-high performance liquid chromatography- Mass Spectrometry (UPLC-MS/MS) was used to measure the levels of lactate in AML12 cells (n=6). **B** IP-WB analysis was performed to detect Trim21, lactylation and ubiquitination levels of Cidec. **C** WB analysis of Cidec protein levels in AML12 cells treated with 20 mM lactate (n=3). **D** qPCR detection of Cidec mRNA expression (n=3). **E** Lipase activity was measured using a commercial assay kit(n=3). **F** BODIPY staining and lipid droplet imaging in AML12 cells after lactate addition (n=50 cells from 5 independent cultures). **G** WB analysis of Ldha and Cidec protein expression in AML12 cells with siRNA-mediated knockdown of Ldha followed by AKG treatment (n=3).**H** IP-WB analysis was performed to detect Trim21, lactylation and ubiquitination levels of Cidec. **I** UPLC-MS/MS was used to measure the levels of lactate in AML12 cells (n=6). **J** Lipase activity was measured using a commercial assay kit (n=5). **K** BODIPY staining and lipid droplet imaging (n=50 cells from 5 independent cultures). Data are presented as mean ± standard deviation. * *p* < 0.05, ***p* < 0.01, ****p* < 0.001, *****p* < 0.0001; ns, not significant. *p* values were determined using an unpaired student's t-test (A, C-F) and One-way ANOVA followed by Bonferroni multiple comparisons test (G, I-K).

**Figure. S9 Lactylation at lysine 124 of Cidec inhibits Trim21-mediated ubiquitination of Cidec in AKG-treated hepatocytes.**

**A** Mass spectrometry analysis of lactylation sites on Cidec. **B** IP-WB detection of ubiquitination levels and interaction with Trim21 for Cidec-WT versus Cidec-K124Q. **C** IP-WB detection of ubiquitination levels and interaction with Trim21 for Cidec-WT versus Cidec-K124R. **D** Lipase activity measured using a biochemical assay kit (n=5). **E** BODIPY staining and lipid droplet imaging (n=50 cells from 5 independent cultures). Data are presented as mean ± standard deviation. * *p* < 0.05, ***p* < 0.01, ****p* < 0.001, *****p* < 0.0001; ns, not significant. *p* values were determined using One-way ANOVA followed by Bonferroni multiple comparisons test (D, E).

**Figure. S10 AKG alters the expression of lipid metabolism-related genes in hepatocytes.**

**A** Principal component analysis (PCA) of liver transcriptomes from mice fed a ND or 2% AKG-supplemented diet (n = 3). **B** Gene Ontology (GO) enrichment analysis of differentially expressed genes from transcriptomic data (n = 3). **C** UPLC-MS/MS was used to determine pyruvate in AML12 cells (n = 6). **D** Cidec lactylation, ubiquitination, and its interaction with Trim21 were assessed in AKG-treated AML12 cells with Pck1 knockdown, followed by treatment with or without lactate. **E** WB analysis of Cidec protein expression in AML12 cells. Data are presented as mean ± standard deviation. **F** Lipase activity measured using a biochemical assay kit (n=5). **G** BODIPY staining and lipid droplet imaging (n=50 cells from 5 independent cultures). * *p* < 0.05, ***p* < 0.01, ****p* < 0.001, *****p* < 0.0001; ns, not significant. *p* values were determined using One-way ANOVA followed by Bonferroni multiple comparisons test (C, E-G).

**Figure. S11 AKG enhances Kdm5 activity to reduce H3K4me3-mediated transcriptional activation of *Pck1*.**

**A** ChIP-seq peaks of H3K9me3, H3K27me3, H3K36me3, and H3K4me3 at the *Pck1* promoter. **B** WB analysis of H3K9me3, H3K27me3, H3K36me3, and H3K4me3 levels in AML12 following AKG treatment (n=3). **C** ChIP-qPCR analysis of H3K4me3 enrichment at the *Pck1* promoter region (n=3). **D** Western blot detection of H3K4me3, Cidec, and Pck1 protein levels in hepatocytes treated with AKG and CPI-455 (n=3). **E** Lipase activity was measured using a commercial assay kit (n=5). **F** BODIPY staining and lipid droplet imaging (n=50 cells from 5 independent cultures). **G** Western blot detection of Kdm5b, H3K4me3, Cidec, and Pck1 levels in AML12 cells treated with AKG and siKdm5b (n=3). **H** Lipase activity was measured using a commercial assay kit in AML12 cells treated with AKG and siKdm5b (n=5). **I** BODIPY staining and lipid droplet imaging in AML12 cells treated with AKG and siKdm5b (n=50 cells from 5 independent cultures). Data are presented as mean ± standard deviation. * *p* < 0.05, ***p* < 0.01, ****p* < 0.001, *****p* < 0.0001; ns, not significant. *p* values were determined using One-way ANOVA followed by Bonferroni multiple comparisons test (B-I).

**Figure. S12 Graphical abstract**

Long-term AKG intake as a dietary supplement could contribute to hepatic steatosis in mice. AKG increases pyruvate and lactate by up-regulating Pck1 expression, leading to increased lactylation of Cidec. Through “lactylation–ubiquitination crosstalk”, AKG inhibits Trim21-mediated ubiquitination and degradation of Cidec, thereby increasing the stability of the Cidec protein.

**Supplementary materials and methods**

**Protein Extraction and Western Blot Analysis**

Proteins were extracted using RIPA buffer (G2002, Servicebio, Wuhan, China) supplemented with phenylmethylsulfonyl fluoride (G2008, Servicebio, Wuhan, China). After adding RIPA buffer and grinding beads (G0101, Servicebio, Wuhan, China) to the liver tissue, the tissue homogenate was obtained by grinding with a homogenizer (KZ-Ⅱ, Servicebio, Wuhan, China) (120 Hz, 120 s). Tissue homogenate or cell lysates were incubated on ice for 15 minutes, with vigorous shaking every five minutes, followed by centrifugation at 16,000 × g for 15 minutes at 4°C. The protein concentration in the supernatant was determined using the BCA Protein Assay Kit (Yeasen, 20201ES86, Shanghai, China). All samples were normalized to equal protein concentrations using RIPA buffer.

An appropriate volume of loading buffer was added, and samples were denatured in a metal heating block at 100°C for 10 minutes. Equal amounts of protein were separated via 10% SDS-PAGE and transferred onto 0.45μm PVDF membranes (WGPVDF45, Servicebio, Wuhan, Chin). Membranes were blocked with 5% non-fat dry milk in TBST and incubated overnight at 4°C with primary antibodies.

Following incubation, membranes were washed with TBST and incubated with horseradish peroxidase-conjugated secondary antibodies for 1 hour at room temperature. Immunoreactive bands were visualized using enhanced chemiluminescence (BMU102, akkine, Wuhan, China) and imaged in a dark room.

Densitometric analysis was performed using ImageJ software (version 1.54f). The expression levels of histone H3 modifications were normalized to total H3 (A22348, ABclonal, Wuhan, China), respectively. Expression of non-histone proteins was normalized to β-Tubulin (A6830, ABclonal, Wuhan, China). All quantitative results were further normalized to the control group. All primary antibodies used in this study were rabbit-derived, and secondary antibodies were goat anti-rabbit.

The following primary antibodies were used:Dgat1 (A6857, ABclonal, Wuhan, China), Cd36 (A5792, ABclonal, Wuhan, China), Acc1(A15606, ABclonal, Wuhan, China), Fasn (A0461, ABclonal, Wuhan, China), Cpt1a(A5307, ABclonal, Wuhan, China), Atgl (A5126, ABclonal, Wuhan, China), Flag (AE092, ABclonal, Wuhan, China), Cidec (12287-1-AP, proteintech, Wuhan, China), Klac (PTM-1401, PTM, Hangzhou, Zhejiang), HA (AE105, ABclonal, Wuhan, China), Trim21 (a1957, ABclonal, Wuhan, China), Kac (9441S, CST, MA, US),Ksucc (PTM-401, PTM, Hangzhou, Zhejiang), Ldha (A18574, ABclonal, Wuhan, China), Pck1 (A22172, ABclonal, Wuhan, China), H3K4me3 (A22224, ABclonal, Wuhan, China), H3K9me3 (A22295, ABclonal, Wuhan, China), H3K27me3 (A2771, ABclonal, Wuhan, China), H3K36me3 (A2366, ABclonal, Wuhan, China), H3 (A2352, ABclonal, Wuhan, China).

The following secondary antibodies were used: HRP-conjugated Goat anti-Rabbit lgG (AS014, Abclonal, Wuhan, China), HRP-conjugated Goat anti-Mouse IgG (AS003, Abclonal, Wuhan, China), HRP-conjugated Mouse anti-Rabbit IgG Light Chain (AS126, Abclonal, Wuhan, China).

**Real-time quantitative Polymerase Chain Reaction (qPCR)**

Total RNA was extracted from liver tissue and cells using TRIzol reagent (R411, vazyme, Nanjing, China) according to the manufacturer’s instructions. Liver tissue was homogenized in a grinding instrument with TRIzol reagent and grinding beads to obtain tissue homogenate. TRIzol reagent was added to the collected cell pellet and left to stand for 5 minutes. Afterward, half the volume of TRIzol reagent was replaced with chloroform, and the mixture was allowed to sit for 5 minutes. The mixture was then centrifuged at 12,000 × g for 10 minutes at 4°C to achieve phase separation. The supernatant was mixed with an equal volume of isopropanol. After centrifugation at 12,000 × g for 10 minutes at 4°C, the RNA pellet was washed twice with 75% ethanol and then dissolved in ddH2O.

One microgram of total RNA was reverse transcribed into cDNA using a commercial reverse transcription kit (G3337, Servicebio, Wuhan, China) following the manufacturer’s protocol. qPCR was performed using SYBR Green Fast qPCR Mix (RK21203, ABclonal, Wuhan, China) on a qPCR detection system (1855201, Bio-Rad, Shanghai, China). Relative mRNA expression levels were calculated using the 2^–ΔΔCt method, with *β-actin* as the internal control.

The following primers were used: *Gck* TgAgCCggATgCAgAAggA/ gCAACATCTTTACACTggCCT, *Adpgk* TCCTgggATggTgTTCCTgAT/ gATCCTggTgAgATCCgAgg, *pgm1* CAgAACCCTTTAACCTCTgAgTC/ CgAgAAATCCCTgCTCCCATAg, *pgm2* AgTgAAgACgCAggCATATCC/ ggCTCCACggTAgAgACgA, *pgm3* AgCAgTgggATgCTATTTATgTC/ TgTCTgCgCTTTCTTgTgAgT, *G6pc1* CgACTCgCTATCTCCAAgTgA/ gTTgAACCAgTCTCCgACCA, *G6pc2* CAggAggACTACCggACTTAC/ TCAACTgAAACCAAAgTgggAA, *G6pc3* CTgAgCgCgggCATCATAAT/ gATTCTTAggATCgCCCAgAAAg, *Pgam* TCTgTgCAgAAgAgAgCAATCC/ CTgTCAgACCgCCATAgTgT, *Fbp1* CACCgCgATCAAAgCCATCT/ AggTAgCgTAggACgACTTCA, *Pfk* ggAggCgAgAACATCAAgCC/ CggCCTTCCCTCgTAgTgA, *Tpi1* CCAggAAgTTCTTCgTTgggg/ CAAAgTCgATgTAAgCggTgg, *Aldo* AgCCTggTCAggTgAAAgC/ ggCCTCCCCAATCTCAgTT, *Gapdh* AggTCggTgTgAACggATTTg/ TgTAgACCATgTAgTTgAggTCA, *Bpgm* ggACCAgAAACTTAACAACgACg/ CAggCTgTgTgAATggACCT, *pgk* ATgTCgCTTTCCAACAAgCTg/ gCTCCATTgTCCAAgCAgAAT, *Pgam1* TCTgTgCAgAAgAgAgCAATCC/ CTgTCAgACCgCCATAgTgT, *Pgam5* ATCTggAgAAgACgAgTTgACA/ CCTgTTCCCgACCTAATggT, *Eno* TgCgTCCACTggCATCTAC/ CAgAgCAggCgCAATAgTTTTA, *Ldha* TgTCTCCAgCAAAgACTACTgT/ gACTgTACTTgACAATgTTgggA, *Ldhb* CATTgCgTCCgTTgCAgATg/ ggAggAACAAgCTCCCgTg, *Aldh3a2* gCggAggATggTgCAAgAg/ CATTgAgTTCACTTTTgCTCAgg, *Aldh2* gACgCCgTCAgCAggAAAA/ CgCCAATCggTACAACAgC, *Acss2* AAACACgCTCAgggAAAATCA/ ACCgTAgATgTATCCCCCAgg, *Dlat* CTTTAgCCTCCAAAgCgAgAg/ AgATTgTAAATgTTCCACCCTgg, *Dld* gAgCTggAgTCgTgTgTACC/ CCTATCACTgTCACgTCAgCC, *Pck1* CTgCATAACggTCTggACTTC/ CAgCAACTgCCCgTACTCC, *Acly* CAgCCAAggCAATTTCAgAgC/ CTCgACgTTTgATTAACTggTCT, *Csl* gggTgggAAAgAACCCCTg/ ggTgACgACATgAgAAggTAgA, *Mdh1* TTCTggACggTgTCCTgATg/ TTTCACATTggCTTTCAgTAggT, *Fh1* TACggAACgTATgCCAATCCC/ ggCCTTCATTATTgCACTAgCA, *CS* ggACAATTTTCCAACCAATCTgC/ TCggTTCATTCCCTCTgCATA, *Aco2* ATCGAGCGGGGAAAGACATAC/ TGATGGTACAGCCACCTTAGG, *Idh1* ATGCAAGGAGATGAAATGACACG/ gCATCACgATTCTCTATgCCTAA, *Idh2* ggAgAAgCCggTAgTggAgAT/ ggTCTggTCACggTTTggAA, *Ogdh* AgggCATATCAgATACgAggg/ CTgTggATgAgATAATgTCAgCg, *Dlst* ggAACTgCCCTCTAgggAgA/ gACgCTACCACTgTTAATgACC, *Sucla2* ACCCTTTCgCTgCATgAATAC/ CCTgTgCCTTTATCACAACATCC, *Suclg1* ggATACgACACgggTCTTACA/ gTggTTCCTCCCACgAgTTTg, *Suclg2* CCCCgAAgATggCTgAACC/ ACCTCCTTTCAAACCgCTATTg, *Sdha* ggAACACTCCAAAAACAgACCT/ CCACCACTgggTATTgAgTAgAA, *Sdhb* AATTTgCCATTTACCgATg9gA/ AgCATCCAACACCATAggTCC, *Sdhc* gCTgCgTTCTTgCTgAgACA/ ATCTCCTCCTTAgCTgTggTT, *Sdhd* TggTCAgACCCgCTTATgTg/ ggTCCAgTggAgAgATgCAg, *β-actin* ggCTgTATTCCCCTCCATCg/ CCAgTTggTAACAATgCCATgT.

**Immunofluorescence**

Cells were seeded on glass coverslips and fixed with 4% paraformaldehyde for 15 minutes at room temperature. After washing with PBS, cells were permeabilized with 0.3% Triton X-100 for 10 minutes and blocked with 5% bovine serum albumin for 1 hour. Cells were then incubated with primary antibodies at 4°C overnight, followed by incubation with secondary antibodies for 1 hour at room temperature in the dark. Nucleus were counterstained with DAPI (BMU107, akkine, Wuhan, China). Fluorescence images were captured using a confocal laser scanning microscope (AX/AX R with NSPARC, Nikon, Tokyo, Japan), and image analysis was performed using ImageJ software.

The following primary antibodies were used: Atgl (A5126, ABclonal, Wuhan, China), Flag (AE024, ABclonal, Wuhan, China), HA (AE105, ABclonal, Wuhan, China).

The following secondary antibodies were used: Cy3 conjugated Goat Anti-Rabbit IgG (GB21303, Servicebio, Wuhan, China), Alexa Fluor® 488-conjugated Goat Anti-Mouse IgG (GB25301, Servicebio, Wuhan, China).

**Oil Red O Staining**

Oil Red O Staining. Oil Red O staining was performed with technical assistance from Servicebio Co., Ltd. (Wuhan, China). Liver tissue was consistently collected from the left lateral lobe to minimize interlobe variability. 8-μm frozen sections were cut from each block at a 50-μm interval, yielding a total of three sections per animal, following the principle of systematic random sampling. Frozen tissue sections were equilibrated to room temperature and fixed in fixative solution for 30 minutes. After fixation, sections were rinsed with 70% ethanol to remove residual fixative. Slides were then incubated in Oil Red O working solution (G1015, Servicebio, Wuhan, China) on a shaker for 10 minutes at room temperature. Following staining, excess dye was removed by washing with 70% ethanol, and slides were rinsed under running tap water for 30 seconds. Nuclei were counterstained with hematoxylin for 2 minutes, followed by an additional rinse in running water to remove excess stain. Slides were sealed using glycerol gelatin mounting medium to prevent fading due to air exposure.

Image Acquisition and Quantification. Stained sections were examined under a light microscope. For each section, five non-overlapping fields were randomly selected by an observer blinded to the experimental groups using a predefined grid pattern. Images were captured at 200× magnification under constant illumination. Quantitative image analysis was performed using ImageJ software (National Institutes of Health, USA). Lipid droplets were segmented using an automated Otsu thresholding algorithm applied uniformly across all images. The area fraction of lipid droplets per field was calculated, and data from all fields and sections were averaged to obtain a single value per animal.

**Biochemical Assays**

The biochemical assay were measured using commercial assay kits according to the manufacturer’s instructions.

**Total Cholesterol (TC) and TG**: They were measured using commercially assay kits (A111-1-1, Nanjing Jiancheng Bioengineering institute, Nanjing, China) (A110-1-1, Nanjing Jiancheng Bioengineering institute, Nanjing, China)according to the manufacturer's instructions. 250 μL of working solution was mixed with 2.5 μL of sample, standard (C_standard), or distilled water (blank), followed by incubation at 37°C for 10 minutes. Absorbance was recorded at 500 nm for TG and at 500 nm for TC using a microplate reader. Concentrations (mmol/L) were calculated as follows:(A_sample − A_blank) / (A_standard − A_blank) × C_standard. For tissue samples, data were normalized to total protein concentration: Concentration (mmol/g protein) = [(A_sample − A_blank) / (A_standard − A_blank)] × C_standard / protein concentration.

**Glucose**: It was measured using a commercially assay kit (F006-1-1, Nanjing Jiancheng Bioengineering institute, Nanjing, China) according to the manufacturer's instructions. Glucose levels were assessed using a similar protocol, with absorbance measured at 505 nm. Glucose concentration was calculated using the following equation: Glucose (mmol/L) = (A_sample − A_blank) / (A_standard − A_blank) × C_standard × dilution factor. The concentration of the standard glucose solution was 5.55 mmol/L.

**Glycogen**: It was measured using a commercially assay kit (KTB1340, Abbkine, Wuhan, China) according to the manufacturer's instructions. Place the liver tissue into Extraction Buffer and boil it in a water bath for 20 minutes. After cooling, add deionized water and centrifuge at 8000g for 10 minutes. Collect the supernatant for further analysis. Add Chromogen to the samples and incubate in a 95°C water bath for 10 minutes. Add Chromogen to deionized water or standard solution to prepare blank and standard wells, respectively, and incubate them at 95°C for 10 minutes. Measure the absorbance at 620 nm.

**Aspartate aminotransferase (AST) / Alanine aminotransferase (ALT)**: It was measured using the commercially assay kit (AST, C010-2-1, Nanjing Jiancheng Bioengineering institute, Nanjing, China; ALT, C009-2-1, Nanjing Jiancheng Bioengineering institute, Nanjing, China) according to the manufacturer's instructions. Briefly, 20 μL of pre-warmed Reagent I was added to both test and control wells, followed by 5 μL of sample in the test wells only. After a 30-minute incubation at 37°C, 20 μL of Reagent II was added to all wells. Then, 5 μL of sample was added to the control wells and incubated for an additional 20 minutes. Reactions were terminated by adding 200 μL of 0.4 mol/L NaOH to each well. Absorbance was measured at 510 nm, and AST/ALT activity (U/L) was determined using a pyruvate standard curve.

**DGAT1 enzymatic activity**: It was measured using a commercially assay kit (HL50830.1, GENMED SCIENTIFICS, Shanghai, China) according to the manufacturer's instructions. Briefly, the culture medium of AML12 cells was discarded, and 3 mL of GENMED Cleaning Solution (Reagent A) was added to fully cover the cell surface. After gentle removal of the solution, the cells were detached using a cell scraper and resuspended in 3 mL of Reagent A. The cell suspension was transferred to a pre-chilled 15 mL conical centrifuge tube and centrifuged at 300 × g for 5 minutes at 4°C.

The supernatant was carefully aspirated, and the cell pellet was lysed in 500 μL of GENMED Lysis Buffer (Reagent B) by thorough mixing. The lysate was then transferred to a pre-chilled 1.5 mL microcentrifuge tube, vortexed vigorously for 15 seconds, and incubated on ice for 30 minutes. After centrifugation at 16,000 × g for 5 minutes at 4°C, 500 μL of the supernatant was collected as the total protein extract. A 10 μL aliquot was used for protein quantification.

To assess DGAT1 enzymatic activity, 20 μL of test sample (containing 500 μg of total protein; sample should be clear) was added to a new cuvette containing GENMED Buffer (Reagent C), Substrate Solution (Reagent E), and either Negative Control Solution (Reagent F) for background control or Reaction Solution (Reagent D) for activity detection, with all reagent volumes following the manufacturer’s protocol. After gentle mixing, the reaction mixture was incubated at 37°C for 30 minutes, followed by the addition of Color Development Solution (Reagent G). After a further 5-minute incubation at 37°C, absorbance was immediately measured at 450 nm using a spectrophotometer. Background values were subtracted to determine net enzymatic activity.

**Fasn activity**: It was measured using a commercially assay kit (ml076653, mlbio, Shanghai, China), following the manufacturer's instructions. Collect the cells and centrifuge them, then resuspend the pellet in Reagent 1. Disrupt the cells on ice using ultrasonic homogenization (300W power, 3 seconds on, 7 seconds off, for a total of 3 minutes). Centrifuge the samples at 12,000g at 4°C for 40 minutes and collect the supernatant for analysis.Pipette 100 μL of the supernatant and sequentially add 20 μL of Reagent 2, 20 μL of Reagent 3, 820 μL of Reagent 4, and 40 μL of Reagent 5. Mix thoroughly and measure the absorbance at 340 nm. Record the absorbance at 30 seconds (A1) and 90 seconds (A2). Subtract A2 from A1 to calculate Fasn activity.

**Lipase (LPS) activity**: It was measured using a commercially assay kit (AKFA006M, Boxbio, Beijing, China), following the manufacturer's instructions. Bacterial or cellular pellets were collected by centrifugation and processed at an approximate ratio of 5 × 10⁶ cells or bacteria per 1 mL of Reagent 1. Samples were lysed by ultrasonic disruption on ice (300 W, 3 s on/7 s off cycles for a total of 3 minutes) and centrifuged at 12,000 × g for 10 minutes at 4°C. The supernatant was collected and maintained on ice.

Prior to the assay, Reagents 1 and 2 were pre-incubated at 37°C for 30 minutes. A spectrophotometer or microplate reader was pre-warmed and set to 710 nm, with toluene used for baseline calibration. In a centrifuge tube, Reagents 1 and 2, sample solution, standard diluent, and distilled water were sequentially added and mixed rapidly. The reaction was conducted precisely at 37°C for 10 minutes, followed by the addition of toluene. After centrifugation at 8,000 × g for 10 minutes at room temperature, the supernatant was collected and mixed with Reagent 3. A second centrifugation (8,000 × g, room temperature, 10 minutes) was performed, and the absorbance of the resulting supernatant was measured at 710 nm to determine LPS activity.

**α-Ketoglutarate (AKG)**: It was measured using a commercially assay kit (S0323S, Beyotime, Shanghai, China), following the manufacturer's instructions. Briefly, serum samples were added to a 96‑well plate, followed by the addition of AKG Assay Buffer to the sample wells. Wells containing only AKG Assay Buffer were used as blank controls. AKG standards were used to generate a standard curve. Amplex Red reaction working solution was added to each well, and the plate was incubated at 37°C for 30 minutes in the dark. The absorbance at 570 nm was then measured. The AKG concentration in the samples was calculated based on the absorbance readings of the blank, the standard curve, and the samples.

**Transcriptome sequencing and analysis**

Total RNA was extracted using TRIzol reagent and stored at −80 °C until analysis. RNA sequencing and bioinformatic analyses were conducted by Novogene. RNA integrity was assessed using the RNA Nano 6000 Assay Kit and the Bioanalyzer 2100 system (Agilent Technologies, CA, US), following the manufacturer’s instructions. Raw reads were processed to remove adaptor sequences, poly-N stretches, and low-quality reads to generate high-quality clean data. Gene expression levels were quantified using FeatureCounts (v1.5.0-p3) to count the number of reads mapped to each gene, and fragments per kilobase of transcript per million mapped reads were calculated accordingly.

Differentially expressed genes (DEGs) were identified using the DESeq2 package in R. Genes with adjusted p-values < 0.01 and |log₂(fold change)| > 0.5 were considered significantly differentially expressed. Functional enrichment analysis of DEGs was performed using the KOBAS 2.0 platform, including Gene Ontology term annotation and Kyoto Encyclopedia of Genes and Genomes pathway analysis.

**Immunoprecipitation**

AML12 cells were washed three times with PBS and then lysed with 500 μL of lysis buffer on ice for 10 minutes. The samples were then centrifuged at 12,000 × g for 10 minutes at 4°C, and the supernatant was collected as the total protein sample. A 1/10 volume of the supernatant was reserved as the input control, while the remaining 9/10 volume was incubated with 20 μL of Anti-Flag magnetic beads (HY-K0207, MCE, NJ, US) at 4°C overnight with gentle rotation to form the protein-beads complex.

The following day, protein-beads complex was captured using a magnetic stand. The supernatant was discarded, and the pellet was washed three times with lysis buffer to remove non-specifically bound proteins. Finally, protein loading buffer was added to the pellet, and the protein-beads complex was dissociated by boiling. The resulting supernatant was collected and subjected to SDS-PAGE analysis.

**Mass spectrum (MS)**

Protein digestion and mass spectrometry were conducted by Beijing Novogene Technology Co., LTD. In brief, gel pieces containing proteins were initially destained using a 1:1 solution of 50 mM ammonium bicarbonate and acetonitrile until fully transparent. The gel was then dehydrated with acetonitrile until it turned white. Subsequently, dithiothreitol (DTT) was added, and the samples were incubated at 37°C for 1 hour in a water bath. Iodoacetamide (55 mM) was then added and incubated for 30 minutes in the dark for alkylation. Afterward, trypsin (0.01 μg/μL) was added to ensure thorough contact with the gel pieces, and digestion was performed overnight at 37°C.

The next day, the digestion supernatant was collected by centrifugation and transferred to a clean tube. The remaining gel pieces were washed with acetonitrile for 5 minutes under vortex agitation, centrifuged, and the supernatant was combined with the previous fraction. The combined solution was vacuum-dried and stored at −20°C until liquid chromatography–mass spectrometry (LC-MS) analysis.

Raw data were analyzed using Spectronaut^®^ 18 software, followed by database searching (Mus musculus uniprot 2024 07 26 swissprot.fasta).

**Bodipy grease drop dyeing**

Cells were cultured to an appropriate density and seeded into confocal culture dishes. After removing the culture medium, cells were briefly rinsed with PBS to eliminate serum residues. A working solution of BODIPY (D3821, ThermoFisher, MA, US) dye was added, and cells were incubated at standard conditions (37°C, 5% CO₂) for 30 minutes. Following incubation, cells were washed 3 times with PBS to remove unbound dye. Lipid droplets were visualized using a confocal fluorescence microscope, with BODIPY emitting green fluorescence.

**Ultra-high performance liquid chromatography- Mass Spectrometry (UPLC-MS/MS)**

Pyruvate and lactate in cell were analyzed using a 3-nitrophenylhydrazine derivatization method. Briefly, for two times, the cell samples were extracted using 600 μL of methanol with an internal standard (10 g/mL of lactate-d3). Following the collection of the supernatant, nitrogen was used to evaporate it. Methanol was used to create the 100 mM 3-nitrophenylhydrazine (3-NPH), 60 mM 1-ethyl-3-(3-dimethylaminopropyl) carbodiimide (EDC), and 2% pyridine (v/v) solutions. 50 μL of solution above was added to the evaporated cell sample, and the mixture was incubated for one hour at 30 °C.50 μL of 0.1% butylated hydroxytoluene (BHT) aqueous solution was added after derivatization, and centrifugation was performed for 10 minutes at 4000 rpm. After that, 100 μL of the supernatant was moved to a new plate for the instrumental analysis. An Atlantis Premier BEH C18 AX Column (2.1 x 50 mm, 1.7 m) was used for the separation process, which was kept at 30 °C. The multiple-reaction monitoring transitions were: Pyruvate, 357.3 -> 137; Lactate, 224.2 -> 137; Lactate-d_3_, 226.9-137. The measurement was performed on an AB SCIEX Triple Quad 6500+ mass spectrometer equipped with an electrospray ionization (ESI) source (AB SCIEX, Singapore). The quantifications were performed against area of internal standards using OS station 2.1 software (AB SCIEX, Singapore).

**siRNA transfection**

The siRNA was synthesized by Wuhan Jinkairei Biotechnology Co., Ltd. siRNA or negative control siRNA was transfected using the non-liposomal PEI transfection reagent (40806ES, Yeasen) according to standard protocols. Cells were seeded 14-18 hours prior to transfection to ensure 50% confluence at the time of transfection. 50 nM of control siRNA (NC) or Cidec-targeted siRNA (siCidec) (sense, 5′-GGACAUCUUGAAACUUAAATT-3′; antisense, 5′- UUUAAGUUUCAAGAUGUCCTT-3′), or Ldha-targeted siRNA (siLdha) (sense, 5′- CAGCAAAGACUACUGUGUATT-3′; antisense, 5′- UACACAGUAGUCUUUGCUGTT-3′), or Pck1-targeted siRNA (siPck1) (sense, 5′- CCUCAGUGAAGACAAAUCCAATT-3′; antisense, 5′- UUGGAUUUGUCUUCACUGAGGTT-3′), or Kdm5b-targeted siRNA (siKdm5b) (sense, 5′- CCUUCGCUUUCAUCCACAA-3′; antisense, 5′- UUGUGGAUGAAAGCGAAGG-3′) were used. After 24 hours of transfection, the cells exhibited normal morphology with no significant cell death observed, and were subsequently used for further experiments.

**Chemicals and reagents**

Antibody Dilution (BMU103, akkine, Wuhan, China), Hieff Trans® PEI MW40000 (40816ES01, yeasen, Shanghai, China), ITSA (HY-100508, MCE, NJ, US), A-485 (HY-107455, MCE, NJ, US), MG-132 (S1748, beyotime, Shanghai, China), CQ (HY-17589AR, MCE, NJ, US), CHX (HY-12320, MCE, NJ, US), Lactate (S108838, aladdin, Shanghai, China), CPI-455 (HY-100421, MCE, NJ, US).

**Plasmid DNA construction and transfection**

pcDNA3.1-Atgl-Flag was cloned into the pcDNA3.1(+)-Flag vector. pcDNA3.1-Cidec-Flag was cloned into the pcDNA3.1(+)-Flag vector. pcDNA3.1-Trim21-HA was cloned into the pcDNA3.1(+)-HA vector. pcDNA3.1- Trim21-His was cloned into the pcDNA3.1(+)-His vector. pcDNA3.1- Ubi-K48-HA was cloned into the pcDNA3.1(+)-HA vector. pcDNA4-Plin2-mCherry-EGFP was cloned into the pcDNA4-mCherry-EGFP vector. All constructions were confirmed by DNA sequencing.

According to the manufacturer's instructions, plasmid DNA was transfected into cells using Polyethylenimine Linear (40816ES01, Yeasen, Wuhan, China).

**Chromatin immunoprecipitation combined with qPCR (ChIP-qPCR)**

ChIP assay was performed following the manufacturer's instructions provided with the commercial ChIP kit (RK20258, Abclonal, Wuhan, China). anti-H3K4me3 (A22224, Abclonal, Wuhan, China) were then used for immunoprecipitation, binding to target proteins. DNA was purified using a commercial DNA extraction kit (RK30100, Abclonal, Wuhan, China) and analyzed by qPCR to quantify assess the binding of *Pck1* promotor.
